# An approach on the implementation of full batch, online and mini-batch learning on a Mamdani based neuro-fuzzy system with center-of-sets defuzzification: Analysis and evaluation about its functionality, performance, and behavior

**DOI:** 10.1371/journal.pone.0221369

**Published:** 2019-09-05

**Authors:** Sukey Nakasima-López, Juan R. Castro, Mauricio A. Sanchez, Olivia Mendoza, Antonio Rodríguez-Díaz

**Affiliations:** Faculty of Chemical Sciences and Engineering, Universidad Autónoma de Baja California, Tijuana, Baja California, México; Newcastle University, UNITED KINGDOM

## Abstract

Due to the rapid technological evolution and communications accessibility, data generated from different sources of information show an exponential growth behavior. That is, volume of data samples that need to be analyzed are getting larger, so the methods for its processing have to adapt to this condition, focusing mainly on ensuring the computation is efficient, especially when the analysis tools are based on computational intelligence techniques. As we know, if you do not have a good control of the handling of the volume of the data, some techniques that are based on learning iterative processes could represent an excessive load of computation and could take a prohibitive time in trying to find a solution that could not come close to desired. There are learning methods known as full batch, online and mini-batch, and they represent a good strategy to this problem since they are oriented to the processing of data according to the size or volume of available data samples that require analysis. In this first approach, synthetic datasets with a small and medium volume were used, since the main objective is to define its implementation and in experimentation phase through regression analysis obtain information that allows us to assess the performance and behavior of different learning methods under distinct conditions. To carry out this study, a Mamdani based neuro-fuzzy system with center-of-sets defuzzification with support of multiple inputs and outputs was designed and implemented that had the flexibility to use any of the three learning methods, which were implemented within the training process. Finally, results show that the learning method with best performances was Mini-Batch when compared to full batch and online learning methods. The results obtained by mini-batch learning method are as follows; mean correlation coefficient $\bar{R}$ with 0.8268 and coefficient of determination $\bar{R^{2}}$ with 0.7444, and is also the method with better control of the dispersion between the results obtained from the 30 experiments executed per each dataset processed.

## Introduction

Currently, different sectors of services and products are requiring systems based on simulation mechanisms for decision making, with the objective of providing greater accuracy, processing of multiple critical variables and in huge volume. As well as the ability to discover hidden relationships, in order to get them valuable insights and knowledge. The capability to build effective solutions that can cope with the complexity intrinsic in data becomes increasingly necessary.

Data analysis increasingly requires powerful tools that can control imprecisions and inconsistencies present in data, which are given by the complex nature of the environment in which they were generated, as well as having the ability to process different volumes of information, for which learning methods are identified, such as Batch, Online and Mini-Batch, which are an excellent alternative to be implemented especially in iterative computational intelligence techniques to help them have a more efficient performance.

Computational Intelligence (CI) is a discipline that has different bio-inspired approaches that are considered excellent universal approximators, the most popular techniques are composed by Fuzzy Logic (FL) [1–2], Artificial Neural Networks (ANN) [3–4], Evolutionary Algorithms (EA) [5–6], Bayesian/Belief networks (BN) [7–8], Particle Swarm Optimization (PSO) [9–10], Kernel methods [11–12], Artificial immune systems (AIS) [13–14], among others, these techniques have the ability to solve multi-objective and non-linear problems.

Excellent results have been obtained when these techniques have been combined, such as learning, adaptation and knowledge representation used together have become in a powerful tool because it is a way to enhance their advantages and overcome the limitations that each technique could individually achieve, this synergy is known as Hybrid Intelligent Systems (HIS). Of the techniques from which greater advantages can be exploited when combined are ANN and FL, since the ANN has the ability to learn from known samples and modeling nonlinear complex relationships, as well as infer hidden relationships what allow a better high volatility of data handling without establishing prior restrictions on inputs, with respect to its distribution; building generalized models giving great results in prediction, classification, and clustering, while FL based on approximate reasoning attempting to mimic human cognitive process, it is easily interpretable and explanatory because of its systems support numeric and linguistic variables [15]. It has applications in different areas in which their performance has given very good results, some case studies are shown as follow:

- Academic management system to model student performance: A neuro-fuzzy adaptive system was proposed. Its architecture is composed of a Neural Network (NN) multi-layer feed-forward and back-propagation as training algorithm and the Sugeno algorithm to fuzzy inference. It is dedicated to decision support and also it allows to measure the effectiveness of teaching-learning methods, competence level, and skills [16].
- Health systems: It was implemented a neuro-fuzzy system with Gaussian defuzzification, the architecture characteristics are composed of four layers. The first layer corresponds to inputs, in the second layer are defined the linguistic terms (Gaussian membership function), the third layer is dedicated to the definition of rules and the last layer are generated the outputs. Its optimization algorithm is Gradient Descent with the back-propagation algorithm for adjustment of parameters (weights). It helps the diagnosis of different diseases, it is a great electrocardiogram signals classifier that help detect ischemic heart disease [17–18].
- Traffic control: It is a generic neuro-diffuse system self-organized, the architecture of this NN is composed of five layers, and its principal's activities execute in each layer are: first layer (defuzzification), second layer (antecedents), third layer (rules base), fourth layer (consequent derivation) and layer fifth (output defuzzification). It is useful for safe and effective traffic management on the roads, also in the definition of tactical maneuvers (lane change, overtaking, vehicle tracking), coalitions forecast, among others [19].

Other Intelligent Hybrid Systems that have also been successful are: Fuzzy support vector machine (FSVM) giving support to class imbalance issues [20], Artificial immune system and genetic algorithm (AIS-GA) aims to automated diagnosis systems [21], Genetic algorithm, and particle swarm optimization (GA-PSO) used to gene selection [22], Deep learning and extreme learning machine (DELM) used in EEG classification [23].

The principal contribution of this study is to define the implementation of three learning methods: Batch, mini-batch, and online. And through experimentation, obtain information that allows us to assess its performance and behavior under distinct conditions, as well as through the implementation of a neuro-fuzzy system, to seek the optimal adjustment of the parameters through learning and the ease of interpretation through Mamdani fuzzy rules.

In this article, we explore different concepts necessary for understanding the of both learning methods and the neuro-fuzzy system implemented, its sections are organized as follow. Hybrid intelligent systems section where will address the generalities of artificial neural networks, fuzzy logic, fuzzy inferences system and learning methods, the section of description of model proposed, followed by the section of explanation of the experiment done and results obtained through regression analysis, section of discussion with regards to performance, accuracy, and stability observed on our model proposed and others existing model and the last section, it is dedicated to highlighting findings discovery and future work directions.

### Hybrid intelligent systems

The combination of different methods of CI provides the opportunity to build HIS, which allow us to extract the advantages that each offers us, for the development of powerful tools that allow solving problems effectively, with learning capabilities and interpretability, achieving with this a good performance and error handling very approximate to human reasoning [24].

CI has elements of learning, adaptation, evolution, and perception, also integrating statistical and probabilistic methods for better support [25]. Hereunder, we will review some techniques and the concepts on which they are based, to have them as a reference when describing the developed model.

#### Artificial neural networks (ANN)

It is part of the set of CI techniques, and it is a discipline that tries to imitate the learning process of the brain. It is also defined as adaptive non-linear and self-organized processing algorithms. It has multiple processing units called neurons, which are interconnected and distributed in different layers of the network, which have the ability to learn based on their inputs and are adapted according to the learning obtained from their environment [26].

**Fig 1:**
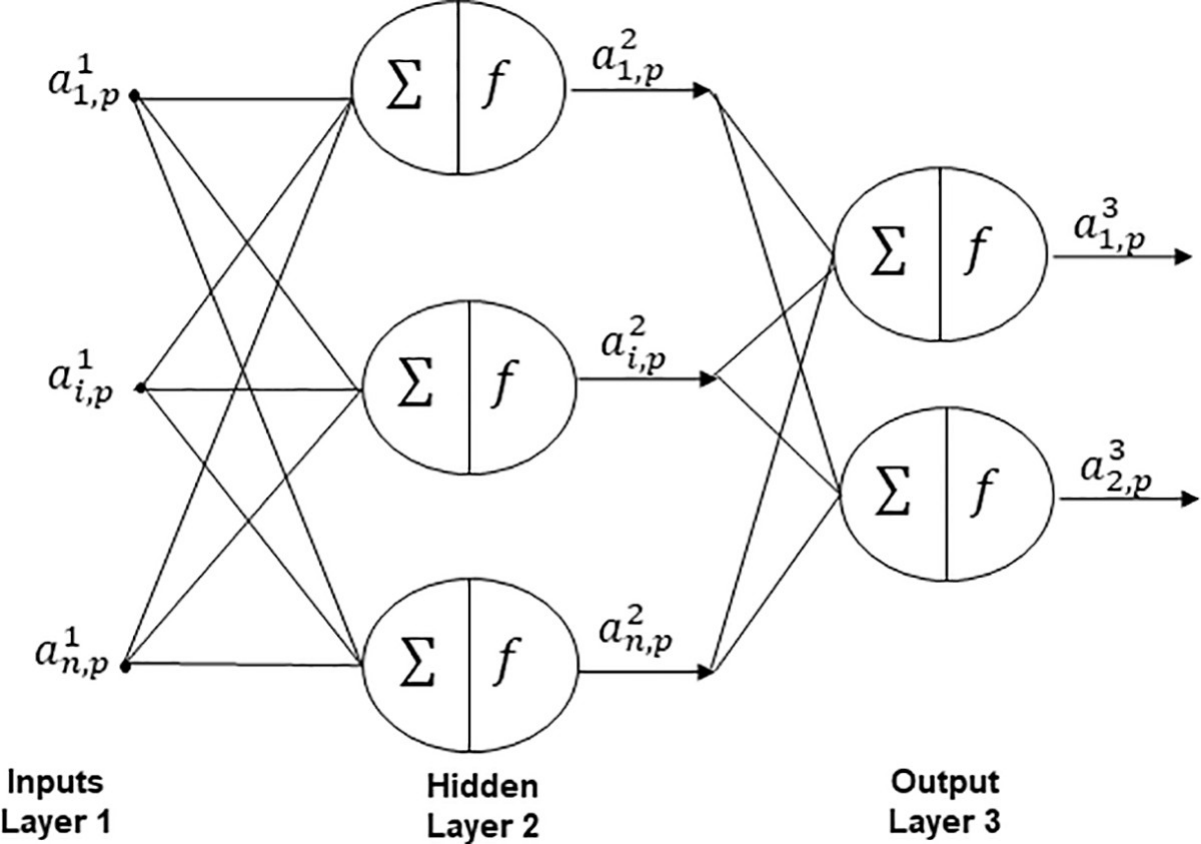
General architecture of artificial neural network.

Their architecture is compound by three major layers wherein each layer does a mapping process from inputs to outputs. The first layer is dedicated to arranging the inputs and it is called the Inputs Layer, and these could be any combination of variables that are important for predicting the output. The output signals between nodes of the layers it is generated through an activation or transfer function. Intermediate layers are known as Hidden Layers and these are considered as essential because endow to ANN to ability to learn the relationships in the data. Finally, in the Output Layer, the results obtained are compared with target sample to know if task has been achieved, these tasks could be classification, clustering, predictions, estimations, among others [27], as can observed in Fig 1.

The functional properties of ANN's are: the mapping process is forward, weights are adjusted iteratively after each training and are stored until the error desired is achieved or the total of epochs are executed. To calculate the error, backpropagation algorithm is implemented that has an inverse direction that feedforward process. To calculate the error, backpropagation algorithm is implemented that has an inverse direction that feedforward process, one of most optimization algorithm used is Gradient Descendant in order to minimize the error [28].

They are characterized by their adaptability, processing in parallel and distributed computing, the processing functions from the input to the output can have a linear, semi-linear or non-linear behavior, the neurons can be defined and distributed according to the needs of the problem [29]. They have the ability to approach several degrees of accuracy and recognize hidden patterns from complex and inaccurate data. They are widely used in problems of control, prediction and classification [30].

Different ANN architectures have been developed, such as, Hopfield Networks, proposed in 1982 by John Hopfield [31]. It is considered one of the simplest because it consists of a single neuron and one layer [32]. Multi-layer Perceptron Networks consists of three layers that are: inputs, hidden and outputs. The hidden layer can be constituted by different numbers of layers and inside them, it can contain different numbers of neurons [33]. Self-organized Maps, also known as Kohonen Maps, are known for their grouping, visualization and classification capabilities. It uses unsupervised and competitive learning [34]. Extreme learning machines are Feedforward Neural Networks. Its learning principle is essentially a linear model. They have a good performance of generalization and learning is faster than networks that use backpropagation training [35]. Convolutional neural networks, like ANN, their neurons self-optimize through learning. One of their significant differences with respect to traditional ANN is that their neurons are organized in three-dimensional layers, which is composed of input dimensionality (height and width) and depth [36]. And Deep learning, considers two key factors: non-linear processing in multiple layers, using supervised and unsupervised learning [37].

The importance of ANN consists of the functional and operational ability stable, it is tolerant to partial, noisy and missing information, in the absence of mathematical representation or model can solve complex problems [28].

#### Gradient descent algorithm

Algorithms based on gradients are one of the most used for the optimization of the error function and parameters adjustment in the training stage of a neural network. They are considered first order, this is referred to how much the function decreases or increases from its first derivative and a specific point of beginning, thus tracing a tangent line over the error surface from the initial point established.

This method has the capability to define a feasible descent directions vector through an iterative process based on information derived from an objective function, where mainly looking adjust parameters (weights) and minimize the model error during its learning process on a spatial of multidimensional inputs to get close a pseudo-optimal solution.

The fundamental components to the implementation of the gradient descent algorithm are:

- The error function is identified as a cost and said cost results to the difference between the estimate response $\hat{y}$ with respect to the response known *y*, one of the error measures most used is Sum of Squares Error (SSE), which is expressed in Eq (1).

$$E=SSE=\sum_{p=1}^{q}{\left(y_{p}-{\hat{y}}_{p}\right)}^{2}\forall p=1,\ldots ,q$$ (1)

- Gradient vector is built through an efficient method known as backpropagation algorithm, in which the partial derivative of the error function with respect to all parameters per each layer are propagated in a form iteratively and inverse to the calculation of the output signals inter-layers processed in the feedforward stage, this could be expressed as shown in Eq (2).

$$g(\xi )=\nabla Ε(\xi )≝{\left[\frac{\partial Ε(\xi )}{\partial \xi_{1}},\frac{\partial Ε(\xi )}{\partial \xi_{i}},\frac{\partial Ε(\xi )}{\partial \xi_{n}}\right]}^{T}\forall i=1,..,n$$ (2)

Where *g*(*ξ*) is the gradient vector to all parameters, *E* is the error function and *ξ* are all parameters that will be adjusted.

- Generalization of delta rule also known as backpropagation learning rule, is the change applied to all parameters to be updated or adjusted, this change is given applying a learning rate (this allows the control of the descent, that is, the adjust velocity) to gradient vector, as is shown in Eq (3).

$$\nabla \xi =-\eta g(\xi )=-\eta \nabla E(\xi )=-\eta \frac{\partial E}{\partial \xi }$$ (3)

Where *∇ξ* is the directional change, −*η* is learning rate and *g*(*ξ*) is the gradient vector, later this directional change is applied to the current parameters, to obtain new adjusted parameters and to continue with the iterative learning process, as is shown in Eq (4).

$$\xi^{new}=\xi^{old}+\nabla \xi$$ (4)

It is easy to implement and shows good results when it comes to nonlinear optimization, however, some of its disadvantages are presented in its performance since it is observed slow and high computing cost when dealing with data sets with high dimensionality.

### Different learning methods based on size and partition of the samples for its processing during the training stage

In order to lighten the computational burden, try to find better global minimums and therefore achieve better convergence, there are different learning methods based on size and partition of the samples, as well as, how the gradient vector will be processed and the learning rule will be applied, these are [38–40]:

- **Batch Training or Full Batch Learning Method:** In this method, the partial derivatives of the error are calculated and accumulated with respect to the parameters for each processed data, that is, it is required to do the entire processing of training sample, in order to build the gradient vector, with which finally, the learning rule that will allow updating or adjusting the parameters of the model can be applied. It is observed in this method that, for samples with small volume (number of instances), its performance is stable and with an acceptable convergence, however, it tends to stagnate in local minimums, besides that when its number of instances increases, its time of computation is excessive, it becomes impractical, it cannot be implemented in a parallel environment and its expense of computation and memory becomes prohibitive. The aforementioned behavior can be expressed as shown in Eq (5):

$$g(\xi ,b)=\nabla E(\xi ,b)=\sum_{p=1}^{q}{\left(y_{p}-{\hat{y}}_{p}\right)}^{2}$$ (5)

Where *y*_*p*_ is the desired output, ${\hat{y}}_{p}$ is the estimated output, and *g*(*ξ*,*b*) the gradient vector constructed from the partial derivative error function *E* with respect to all its parameters *ξ* and bias *b*, finally the Eq (6) represents the learning rule:

$$\xi^{new}=\xi^{old}-\eta g(\xi ,b)$$ (6)

Where *η* is the learning rate, *ξ*^*old*^ corresponds to the current parameters and to which the change obtained from the learning rate and gradient vector will be applied, to generate its update, represented by *ξ*^*new*^.

The following pseudo-code tries to represent the functional behavior of this method:

1. while epoch number does not reach its defined maximum
2.     for-each data in training sample
3.         The gradient is calculated for all parameters *g*(*ξ*_*p*_,*b*) ∀*p* = *number of data*
4.         The obtained gradient is accumulated $g(\xi ,b)=\sum_{p=1}^{q}g(\xi_{p})\forall p=1,..,q$
5.     end
6. end
7. The cumulative gradient and the learning rate are used to update the parameters
8. *ξ*^*new*^ = *ξ*^*old*^−*η*_*g*_(*ξ*,*b*)
9. end

- **Online training or online learning method**: in this method, the updating of the design parameters is done every time a data is processed, that is for each data that is in the sample at the same epoch. This type of procedure leads to the calculation of an approximate gradient, its main advantage being the increase in speed, it is adaptable because it is not based on the distribution of its data, unlike the batch learning method, it greatly decreases the load in memory of data and also the cost of computing them, can work in real-time environments, however, their descent is a bit unstable, due to the noise implicit in each data of the processed sample (high variability), which could be beneficial, since that their jumps could be interpreted as potential better local minimums and even achieve a global minimum. Said behavior can be represented as shown in Eq (7):

$$\xi^{new}=\xi^{old}-\eta g(\xi ,b,x^{\left(p\right)},y^{\left(p\right)})$$ (7)

Where *g*(…) represents the gradient vector, and the parameters within the function are *ξ* design parameters, *b* bias, *x*^(*p*)^ inputs, *y*^(*p*)^ desired output and *p* represents the index or position of the data to be processed.

The following pseudo-code tries to represent the functional behavior of this method:

1. while epoch number does not reach its defined maximum
2.     for-each data in training sample
3.         The gradient is calculated for all parameters *g*(*ξ*,*b*,*x*^(*p*)^,*y*^(*p*)^)∀
4.                 *p* = number of data
5.         The obtained gradient and the learning rate are used to update the parameters
6.             *ξ*^*new*^ = *ξ*^*old*^−*ηg*(*ξ*,*b*,*x*^(*p*)^,*y*^(*p*)^)
7.     end
8. end

- **Mini-batch training o mini-batch learning**: This method improves on difficulties presented both in full batch and online learning method, on one side, it tries to reduce the high variability generated when calculating for each one data the gradient vector, this behavior is shown when Online learning method is executed, or well another behavior that could be presented is when the whole sample is used to build the gradient vector as is the case of the full batch learning method what causes a high cost computational when loading in memory the full sample to be processed. Mini-Batch learning method tries to take advantage of both of them methods, through a partitioning of the data sample into smaller data samples, this combination can lead to a stable descent, greater velocity and reduction of variability, it is also widely parallelizable, so it can be executed in a distributed manner, recommended for use in big data or deep learning environments.

This method is the result of the accumulation of its partial derivatives for each processed mini-batch, where the gradient vector is constructed from the error function averaged in between the defined mini-batch, with respect to all the parameters to be updated or adjusted. Said behavior can be represented under the following Eq (8):

$$g(\xi ,b,x^{\left(p:bs\right)},y^{\left(p:bs\right)})=\nabla E(\xi ,b,x^{\left(p:bs\right)},y^{\left(p:bs\right)})=\sum_{p=1}^{bs}(y^{\left(p\right)}-{\hat{y}}^{\left(p\right)}{)}^{2}$$ (8)

Where *g*(…)represents the gradient vector, *ξ* parameters to be updated or adjusted, *b* bias, the following parameters correspond to each mini-batch to process, these are; *x*^(*p*:*bs*)^ inputs, and *y*^(*p*:*bs*)^ desired output, p index or position of the data in the mini-batch and bs represents the size of the mini-batch to be processed. Finally, Eq (9) represents the learning rule which will allow the adjustment or update of parameters.

$$\xi^{new}=\xi^{old}-\eta g(\xi ,b,x^{\left(p:bs\right)},y^{\left(p:bs\right)})$$ (9)

The following pseudo-code tries to represent the functional behavior of this method:

1. while epoch number does not reach its defined maximum
2.     while number of mini-batch does not reach total limit of mini-batch
3.         for-each data in current mini-batch training sample
4.             The gradient is calculated for all parameters
5.                         o*g*(*ξ*,*b*,*x*^(*p*:*cbs*)^,*y*^(*p*:*cbs*)^)∀*p* = number of data;
6.                     *cbs* = current batch size
7.         end
8.         The obtained gradient of mini-batch processed is accumulated
9.                     $g(\xi ,b,x^{\left(p:bs\right)},y^{\left(p:bs\right)})=\sum_{p=1}^{bs}g(\xi ,b,x^{\left(p:bs\right)},y^{\left(p:bs\right)})$
10.                       ∀*p* = 1,..,*q*;*bs* = batch size
11.                   end
12.           The cumulative gradient and the learning rate are used to update the parameters
13.                               *ξ*^*new*^ = *ξ*^*old*^−*ηg*(*ξ*,*b*,*x*^(*p*:*bs*)^,*y*^(*p*:*bs*)^)
14.     end

#### Fuzzy logic system (FLS)

From 1920, Lukasiewiscz's spoke about the fact that the values in the logical systems were nothing more than a logic with continuous values [41]. By 1965, Zadeh achieved to crystallize his idea of Cointensive Indefinability which he said was a qualitative measure of proximity of meanings of precision, from which he created his concept of degree of membership, which has been one of the fundamental bases for the development of the theory of fuzzy sets [42,43].

FL was introduced in 1975 by Zadeh in a paper titled “Fuzzy logic and approximate reasoning”. Its inspiration is based on the reasoning of the human mind that is approximate rather than exact, giving more importance to the meaning than to the precision of the resultant information [44]. For example, when an object is about to fall on a person's head, the important information for this person is to know that an object will fall on him and not the weight, shape, trajectory, and speed in which this object will fall on him.

The definition of a complex behavior cannot be expressed precisely, instead, we need a system that can tolerate inaccuracies, incomplete information, perceptions, experiences, and judgments, for this reason, FL requires concepts such as fuzzy sets, linguistic variable, and if-then rules to build a robust system.

A fuzzy set is defined as a set continuous function in the universe of the discourse of X, whose domain is defined with values [0,1], in this context, classical binary logic can be seen as a particular case of Fuzzy Logic (FL). Such a continuous function is known as Membership Functions (MF), it is denoted as *μ*_*A*_(*x*) and is called a type-1 MF, where A is a fuzzy set of continuous universes of X, and can be expressed as (10):

$$A=\{x,\mu_{A}(x)\vee x\epsilon X\},\;in\;which\;0\leq \mu_{A}(x)\geq 1$$ (10)

The value of *μ*_*A*_(*x*) is called the degree of membership, or membership grade, of x in A. The distributions most commonly used for MFs are triangular, trapezoidal, piecewise linear, Gaussian, and bell-shaped.

Linguistic variables allow to represent knowledge in approximate reasoning. Values in this variable are words or sentences in natural language. These variables are characterized by a quintuple, expressed as (*X*,*T*(*X*),*U*,*G*,*M*), where *X* is the name of linguistic variable, *T*(*X*) is the collection of linguistic values, *U* is the universe of discourse (or numeric domain subjacent), G free-context grammar and M is a semantic rule that associate each linguistic value with its meaning.

The representation of knowledge is implemented in a proposition in a form of rules if-then and it is known as fuzzy rules. These rules are expressed as *IF*⟨*antecedents*⟩*THEN*⟨*consequent*⟩ both antecedent and consequent are fuzzy propositions that contain linguistic variables. Since fuzzy sets do not have a finite set of possibilities defined for each input, it is necessary to express its operators as functions for all probable fuzzy values. These operators are expressed for all A and B fuzzy set as:

- Intersection (operator AND): its generalized form is known as T-norm (11).
  $$\mu_{A\cap B}(x)=min(\mu_{A}(x),\mu_{B}(x))\forall x\in X$$ (11)
- Union (operator OR): its generalized form is known as T-conorm (12).
  $$\mu_{A\cup B}(x)=max(\mu_{A}(x),\mu_{B}(x))\forall x\in X$$ (12)
- Complement (operator NOT): where the fuzzy set $\overset{´}{A}$ denote de complement of fuzzy set *A* (13).
  $$\mu_{\overset{´}{A}}(x)=1-\mu_{A}(x)\forall x\in X$$ (13)

T-norm and T-conorm are considered as generalized disjunction and conjunctions respectively and are known as fuzzy implication. The implication is one of the major connectives in any logical systems, and it has a very serious influence on the performance of the systems in which fuzzy logic techniques are employed [45].

#### Fuzzy inference system (FIS)

It is a framework based on concepts of fuzzy logic, fuzzy set theory, and fuzzy rules, that have been success in application areas, such as control, support decision, identification systems, among others. Its strength relies on it is capable of handle linguistic concepts (model natural language), it is considered as universal approximator and it is able to perform nonlinearly mapping between inputs and outputs [46].

**Fig 2:**
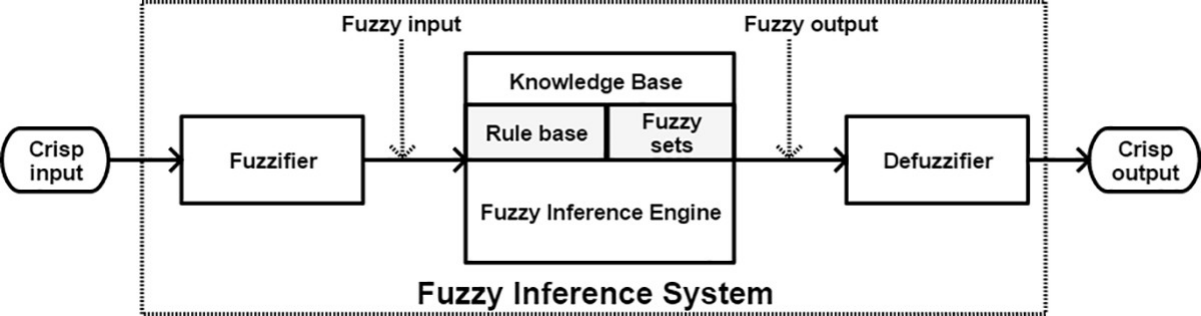
General architecture of a FIS.

The general architecture of a FIS is based on the follows components; fuzzifier, fuzzy inference engine, and defuzzifier as shown in Fig 2 [47–48].

The details of each component FIS are described below [47]:

- Fuzzifier is in charge to convert crisp values of the universe of discourse and determine of the membership degree of these inputs to the associated fuzzy set, through mathematical procedures. For example, let A and B two fuzzy sets and X the universe of discourse, the fuzzification process take the values received *a*,*b*∈*X*, and then produce a membership degree, that can be expressed as follows (14):
  $$\mu_{A}(a),\mu_{A}(b),\mu_{B}(a)\wedge \mu_{B}(b)$$ (14)
- Fuzzy inference is the process where FL is used through its fuzzy rules, membership functions and operators of fuzzy implication, with the aims of mapping inputs values to outputs. The flow of this process is as follow; the fuzzified inputs are mapping to a rule base, in this phase, the antecedent part of the proposition, input sets can be combined through fuzzy operators to generate a compound fuzzy proposition, and the consequent part are determined by degrees of membership in inputs sets and its relationships. The rules base can be seen as follows (15):
  $$R^{k}=IFx_{1}isA_{1}^{k}\wedge x_{2}isA_{2}^{k}THENy_{1}isB_{1}^{k},y_{2}isB_{2}^{k}$$ (15)
- Aggregation, in this process, all fractional membership functions resulting from consequent part are combined, with the objective to obtain a consolidate fuzzy set. In this phase, a singleton value is determined for each $y_{i}\in B_{i}^{k}$, generality using the max operator, this can be expressed as follows (16):
  $$\beta_{i}=\underset{\forall k}{max}(\alpha_{k_{i}})$$ (16)
  Where $\alpha_{k_{i}}$ is the rule firing of each output *y* in the consequent.
- Defuzzifier, this is the process in charge to convert a fuzzy set to crisp number as output of a fuzzy system, this value can be used in expert system to make a decision or in a controller to exercise action. In a fuzzy system with more than output variable, the defuzzification processes are by each output. There are many different of defuzzification methods, one of them is a variation of the max criterion method, where the popular method in this category is MOM (Mean of Maxima), in this method, the final output value is calculated by averaging all output values with the highest membership values. The equation that represent this behavior is (17):
  $$Ζ=\frac{\sum_{i=1}^{l}x_{i}}{l}\forall x_{i}\epsilon M$$ (17)
  Where *M* is the set that contains all maximum membership values, these values are represented by *x*_*i*_, and *l* is the cardinality of the set *M*.
  Other popular methods are those based on centers:
  Center of Sums (COS), in this method, first calculate the geometric center of area for each membership. The equation that represent this behavior is (18):
  $$Z=\frac{\sum_{i=1}^{n}{CoA}_{i}{area}_{i}}{\sum_{i=1}^{n}{area}_{i}}$$ (18)
  Where *CoA*_*i*_ is the geometric center of area of the scaled membership function in the *i*^*th*^ rule, that there fractioned by firing strength identified, *n* is the number of scaled membership functions, and *area*_*i*_ is the area of the scaled membership function *n*.
  Center of sets (COS), in this method, for each rule consequent a singleton centroid is located, as well as, the firing level is necessary. The equation that represent this behavior is (19):
  $$y_{cos}(x^{\prime })=\frac{\sum_{l=1}^{M}c^{l}f^{l}(x^{\prime })}{\sum_{l=1}^{M}f^{l}(x^{\prime })}$$ (19)
  Where *c*^*l*^ is the centroid, *l* is the *l*^*th*^ consequent set, and its firing level represented by *f*^*l*^ given by a fuzzy value contained in *x*′.
  Other methods based on centroid is Center of gravity (COG), in this method, COG is calculated over a series of points continuums in the scaled membership function and finds a representative point of COG from fuzzy set. This method can be expressed mathematically as follow (20):
  $$COG=\frac{\int_{a}^{b}\mu_{A}(x)xdx}{\int_{a}^{b}\mu_{A}(x)dx}$$ (20)
  Where *A* is the sub-area from fuzzy set evaluated, *a*,*b* is the interval of the sub-area of *A*, and *x* is the sample of values in this interval from sub-area of *A*.
  There are other more defuzzification methods, but only showed the based-on centers and centroids, because the architecture neuro-fuzzy system proposed was based in el defuzzification method on center-of-sets.

The two most used FIS are the Mamdani-type and the Sugeno-type, and their most relevant differences are in the output, since Mamdani-type generate a fuzzy sets and Sugeno-type are linear functions or constants in the consequent.

## Materials and methods

### Description of proposed model

The design and implementation of a Mamdani-based neuro-fuzzy system with center-of-sets defuzzification was made, that provide flexibility to handle inputs and outputs multiple, as well as, also can use any of the different learning methods, such as, batch, online or mini-batch, since the existing neuro-fuzzy systems only support batch learning method. As mentioned, what this study seeks is to establish a reference with respect to the implementation of different learning methods based on size and partition of the samples, the accumulation or not of gradient vector calculated and the form that the parameters will be updated or adjusted during the training stage. For this reason, we opted for the development of a neuro-fuzzy system that would allow us to experiment with all three learning methods, full batch, online and mini-batch.

#### Description of the architecture of the Mamdani based neuro-fuzzy with center-of-sets defuzzification

The proposed neuro-fuzzy system was defined as feedforward for the calculation of the output signals, where each of its layers are defined as follows; the zero layer corresponds to the inputs (which can range from ***1 to*** *n*), the first hidden layer consists of adaptive nodes, they are considered adaptive since it contains design parameters that will be adjusted through the iterative process backpropagation, since this is where the error is calculated from the output and it is propagated between its layers of an inverse form to the feedforward, within each node, a Gaussian function has been defined and its adjustable parameters are; its means and standard deviations, as well as the previous establishment of the rules number (*r*) that will correspond to the number of nodes that will contain both layer one and layer two.

**Fig 3:**
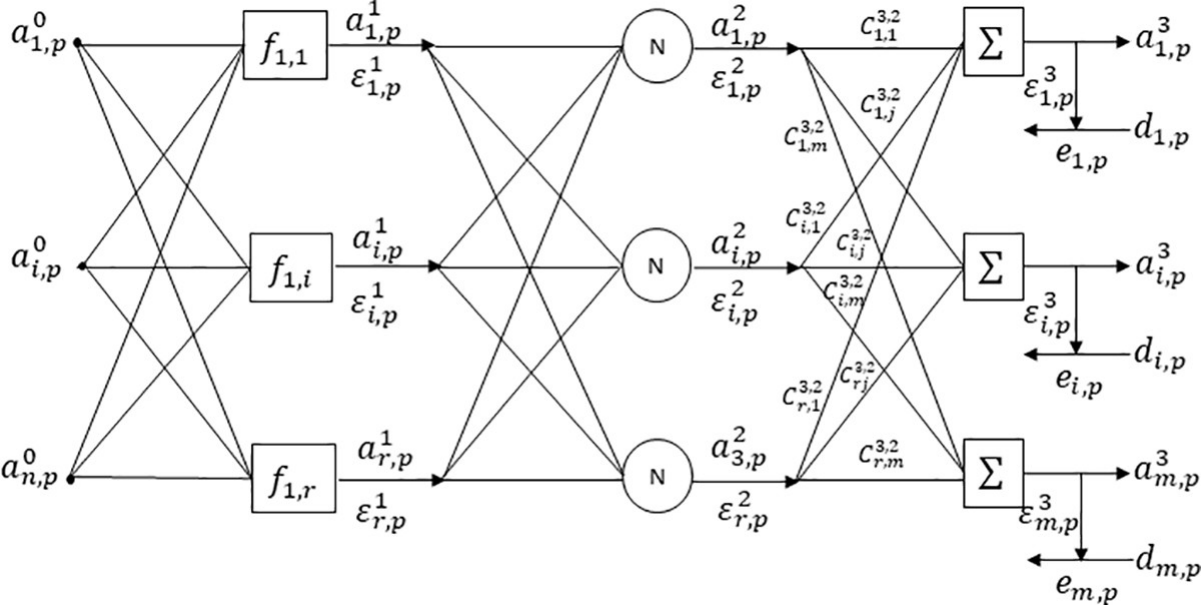
General architecture of the proposed Mamdani based neuro-fuzzy system with center-of-sets defuzzification.

The output signals of the first hidden layer will be the inputs to the second hidden layer, which consists of fixed nodes and where a normalization of said signals will be processed, finally to generate the calculation of the output signals of the last layer that will be defined by *m* outputs, both the signals of the output of the hidden layer two and its centroids (the latter considered also design parameters, so they will also be adjusted during the training) will be required, all this behavior can be observed in the (Fig 3).

The above defines the elements and behavior of the neural network for the optimization of the design parameters, on the other hand, it also makes the definition of the elements and behavior of the fuzzy part of this system. We begin by defining the knowledge base based on Mamdani, as shown in the generic rule (21).

$$R^{k}:IFx_{1}isF_{1}^{k}\wedge x_{i}isF_{i}^{k}\wedge x_{n}isF_{n}^{k}THENy_{1}isC_{G_{1}^{k}},y_{i}isC_{G_{i}^{k}},y_{m}isC_{G_{m}^{k}}$$ (21)

Where *R*^*k*^ corresponds to the *kth* rule, the antecedent part is defined by its entries represented by r $x_{i}^{n}\forall i=1,\ldots ,n$, the definition of its firing forces represented by $F_{i}^{k}\forall i=1,\ldots ,k$, the consequent part defined by their outputs $y_{i}^{m}\forall i=1,\ldots ,m$ and its generic centroids represented by $C_{G_{m}^{k}}\forall m=1,\ldots ,k.$ Hereunder, each one of the layers defined in the architecture of the neuro-fuzzy system proposed is detailed:

- Layer 0: in this layer is to find the input matrix (22), which are used to calculate the firing force. The sub-index *i*_0_ corresponds to the entries in layer zero and *p* it is the sub-index of the data for each entry.
  $$a^{0}=[a_{i_{0},p}^{0}]\forall p=1,..,q;i_{0}=1,\ldots ,n$$ (22)
- Layer 1: in this layer, the firing force is calculated from the inputs $a_{i_{0},p}^{0}$ and the adjustable design parameters during the iterative backpropagation process, are; $m_{i_{1},i_{0}}$ (mean) and $\sigma_{i_{1},i_{0}}$ (standard deviation). The adaptive nodes of this layer contain a Gaussian function and their definition is shown in the Eq (23).
  $$a_{i_{1},p}^{1}=exp\left[\frac{-1}{2}\{\sum_{i_{0}=1}^{n}{\left(\frac{a_{i_{0},p}^{0}-m_{i_{1},i_{0}}}{\sigma_{i_{1},i_{0}}}\right)}^{2}\}\right]$$ (23)
  $$\forall p=1,..,q;i_{1}=1,\ldots ,r$$
  Where sub-index *i*_0_ corresponds to the inputs of layer 0, sub-index *i*_1_ corresponds to neurons in layer 1, *r* is the rules number in the layer, *p* is the input data index and *q* is the total number of data.
- Layer 2: in this layer, firing forces are normalized (24), from the output signals of the previous layer.
  $$a_{i_{2},p}^{2}=\frac{a_{i_{1},p}^{1}}{\sum_{i_{1}=1}^{r}a_{i_{1},p}^{1}}$$ (24)
  $$\forall i_{2}=1,\ldots ,r;\;i_{1}=1,\ldots ,r;\;p=1,..,q$$
  Where $a_{i_{1},p}^{1}$ is the output signal of the layer 1.
- Layer 3: in this layer, the output signals are obtained $a_{i_{3},p}^{3}$, from the output signal of the previous layer $a_{i_{2},p}^{2}$ and the centroids that are part of the design parameters $C_{i_{3},i_{2}}$ (25).
  $$a_{i_{3},p}^{3}=\sum_{i_{3}=1}^{m}C_{i_{3},i_{2}}a_{i_{2},p}^{2}\forall i_{2}=1,\ldots ,r;i_{3}=1,\ldots ,m$$ (25)
  This whole trajectory is known as the feedforward method, once reached the last layer, it begins the backpropagation process to calculate the error partial derivates with respect to all design parameters:
- Error calculation: obtained from the difference of the desired output and the estimated output signal (26).
  $$e=T-a_{i_{3},p}^{3}$$ (26)
- Error calculation squared by each data: of the obtained, the error is individually squared (27).
  $$E_{p}=\sum_{i_{3}=1}^{m}e_{i_{3},p}^{2}$$ (27)
  $$\forall i_{3}=1,\ldots ,m$$
- Sum of the squared errors: a summation is made to obtain a total error, from the error by each data raised to the square (28).
  $$E=SSE=\sum_{p=1}^{q}E_{p}$$ (28)

After the feedforward routine was finished, the iterative process backpropagation begin, this is the procedure to find the gradient vector (33). Its inverse trajectory is done as follows:

- Layer 3: the error is calculated $\varepsilon_{i_{3},p}^{3}$ (29), this is the derivative of the error measure *E*_*p*_.
  $$\varepsilon_{i_{3},p}^{3}=-2e_{i_{3},p}$$ (29)
  $$\forall p=1,\ldots ,q;i_{3}=1,\ldots ,m$$

In this same layer, the partial derivative of the error with respect to the centroid is evaluated $C_{i_{3},i_{2}}$ and the output of layer 2 (30).

$$\frac{\partial^{+E}}{\partial C_{i_{3},i_{2}}}=\sum_{p=1}^{q}\frac{\partial^{+E_{p}}}{\partial C_{i_{3},i_{2}}}=\varepsilon_{i_{3},p}^{3}.a_{i_{2},p}^{2}$$ (30)

$$\forall p=1,\ldots ,q;i_{3}=1,\ldots ,m;i_{2}=1,\ldots ,r$$

- Layer 2: the error is calculated $\varepsilon_{i_{2},p}^{2}$, from the centroids and error of layer 3 (31).
  $$\varepsilon_{i_{2},p}^{2}=\sum_{i_{3}=1}^{m}C_{i_{2},i_{3}}\varepsilon_{i_{3},p}^{3}$$ (31)
  $$\forall p=1,\ldots ,q;i_{3}=1,\ldots ,m;i_{2}=1,\ldots ,r$$
- Layer 1: the error $\varepsilon_{i_{1},p}^{1}$ is calculated from the difference between the error of the previous layer $\varepsilon_{i_{1},p}^{2}$ minus the error product of the same layer and the output signal of the previous layer $a_{i_{2},p}^{2}$ between each of the output signals of the current layer $a_{i_{1},p}^{1}$ (32).
  $$\varepsilon_{i_{1},p}^{1}=\frac{\varepsilon_{i_{1},p}^{2}-\sum_{i_{2}=1}^{r}{\varepsilon_{i_{2},p}^{2}a}_{i_{2},p}^{2}}{\sum_{i_{1}=1}^{r}a_{i_{1},p}^{1}}$$ (32)
  $$\forall p=1,\ldots ,q;i_{1}=1,\ldots ,r$$

The derivatives of the mean parameters were calculated (33) and standard deviation (34), same that will serve to build the gradient vector, to finally update the design parameters with the new directional vector.

$$\frac{\partial^{+E}}{\partial m_{i_{1},i_{0}}}=\sum_{p=1}^{q}\frac{\partial^{+E_{p}}}{\partial m_{i_{1},i_{0}}}=\varepsilon_{i_{1},p}^{1}a_{i_{1},p}^{1}\frac{a_{i_{0},p}^{0}-m_{i_{1},i_{0}}}{\sigma_{i_{1},i_{0}}^{2}}$$ (33)

$$\frac{\partial^{+E}}{\partial \sigma_{i_{1},i_{0}}}=\sum_{p=1}^{q}\frac{\partial^{+E_{p}}}{\partial \sigma_{i_{1},i_{0}}}=\varepsilon_{i_{1},p}^{1}a_{i_{1},p}^{1}\frac{{\left(a_{i_{0},p}^{0}-m_{i_{1},i_{0}}\right)}^{2}}{\sigma_{i_{1},i_{0}}^{3}}$$ (34)

$$\forall i_{0}=1,\ldots ,n;i_{1}=1,\ldots ,r;p=1,\ldots ,q$$

Once the partial derivatives of the mean, standard deviation and centroids parameters with respect to the error are obtained, the gradient vector is built, and a learning rate is applied (35) to generate the directional change with it which will update or adjusted all parameters (36).

$$\nabla \xi =-\eta \nabla E(\xi )=-\eta \frac{\partial E}{\partial \xi }$$ (35)

$$\xi^{new}=\xi^{old}+\nabla \xi$$ (36)

Where *ξ*∈{*σ*,*m*,*C*} is the vector of all design parameters of the Mamdani-based neuro-fuzzy system with center-of-sets defuzzification, and −*η* corresponds to the change in the learning rate which is part of the training parameters.

#### Hyperparameters and functionality description

As is known, hyperparameters are those that must be configured prior to the execution of training, although there are recommendations to choose their appropriate values, there is nothing conclusive, as in the case of the rate of learning, it is known that a learning rate with very low values will lead to a slow and computationally expensive training, since it will require many iterations to be able to approach a quasi-optimal solution and depending on the complexity of the dataset, it could never be achieved, or, if the established value is very high, it will tend to diverge the solution area. In the case of the experimentation carried out, to palliate this problem, for each dataset analyzed, the best values for each hyperparameter were located, establishing a range of values and searching among them, those that would provide better results with respect to the minimization of the error function. The hyperparameters required for the Neuro-Fuzzy system that was proposed are:

- **Training parameters**, these parameters are defined in order to control training duration, as described below:
  ■ **Total epochs number** is the maximum limit of iterations that will be carried out during training.
  ■ **Goal error**, the minimum value of the ideal error that could reach.
  ■ **Learning rate** (*η*), this parameter allows us to determine how fast or slow the gradient vector moves towards obtaining the optimal parameters.
  ■ **Momentum** (*mc*), fraction of the change in parameters that allows smoothing the oscillation in the trajectory, either increasing or decreasing the parameters change in each iteration [49].
  ■ **Maximum validation failures number** is the maximum limit of failures allowed in the validation process.
  ■ **Maximum error increase**, refers to the maximum allowed limit of the calculated error
  ■ **Minimum gradient limit** is the minimum value of the norm of the calculated gradient vector.
  ■ **Decrease rate**, this value is established, to decrease the learning rate to this defined proportion, in case the calculated error is greater than the maximum increase established.
  ■ **Increase rate**, this value is established to increase the learning rate to this defined proportion, in case the calculated error is less than previously calculated.
  ■ **Rules number** (*r*), this value is established, to indicate the rules number with which the fuzzy part will count, this parameter will be equivalent to the number of neurons that will contain layer 1 and 2 of the network.
- **Design parameters** are those that will be adjusted in each execution of the training until reaching the optimum. These parameters are; *mean*, *standard deviation and centroid*. Its initial values are obtained from the dataset to be processed, thus generating a matrix with *r*×*n* dimension, where *r* is the rules number and *n* is the inputs number.
- Three main functional processes are identified, to carry out the learning, these are:
- **Initialization**: In this first process, the initial change of the design parameters is calculated as is represented in Eq (37), it is required since in the training the optimization function of the Gradient Descendent with Momentum and Adaptive Learning Rate will use it.
  $$\nabla \xi_{prev}=-\eta \frac{\partial E}{\partial \xi }$$ (37)
- **Training:** It starts calculating the Gradient Descendent with Momentum and Adaptive Learning Rate (38):
  $$\nabla \xi_{now}=mc\nabla \xi_{prev}-(1-mc)\eta g(\xi )$$ (38)
  It is in this process, where the learning method to be used is established, which will define the way in which you will be sending the data to generate the gradient vector and the calculation of the error.
  In order to control the velocity and direction of gradient descent, the following heuristics were implemented as shown in the following pseudo-code:

1. While epoch number does not reach its defined maximum
2.     Gradient descent with momentum and adaptive learning rate is calculated
3.             *∇ξ*_*now*_ = *mc∇ξ*_*prev*_−(1−*mc*)*ηg*(*ξ*)
4.     Temporal update parameters are calculated *ξ*^*temporal*^ = *ξ*^*old*^+*∇ξ*_*now*_
5.     Current error is calculated with the change applied to the parameters
6.     If (current error/previous error) is greater than maximum error increase parameter then
7.         Learning rate is decreased *η* = η*decreaserateparameter
8.         The gradient vector is updated applying the new learning rate *g*(*ξ*) = η*g(*ξ*)
9.         Else
10.             If current error is smaller than previous error then
11.                 Learning rate is increased *η* = η*increaserrateparameter
12.             end
13.             Parameters are updated with the change calculated as in line 4, but now in permanent form
14.                     *ξ*^*new*^ = *ξ*^*old*^ + *∇ξ*_*now*_
15.             Previous error is replaced by current error
16.             Gradient vector is recalculated with the new parameters
17.         End
18.     End

The following criteria were also considered, which allow deciding the moment in which the training should be interrupted, this is:

■ When the epochs number processed is equal to the training parameter defined as total epochs number.
■ When the calculated error is less than or equal to the training parameter defined as goal error.
■ When the norm of the gradient vector is less than the training parameter defined as the minimum gradient limit.
■ When the accumulated validation failures number is greater than the training parameter defined as the maximum validation failures number. Lastly is calculated from the validation data sample, the calculation of the error (using the design parameters that were previously adjusted in the training), it is validated if the current error is greater than the previous error, in this case it is increased a counter of validation faults, and if this counter becomes greater than this parameter, then training is interrupted.

Finally, the error function that is sought to be minimized during the training process was defined as Sum of the squared errors, this is a validation metric which allows to verify the progress of the training in the direction of the optimal convergence.

## Results and discussion

In this section, the experimentation methodology and the regression analysis of the results obtained are presented. The objective of this analysis is to carry out a comparative study of the performance of each of the exposed learning methods (batch, online and mini-batch), executed on a proposed Mamdani based neuro-fuzzy system with center-of-sets defuzzification.

Different datasets were used to carry out experimentation, their descriptive characteristics being those found in Table 1.

**Table 1:** Descriptive characteristics from used datasets in experimentation [50–51].

Datasets	Dataset description	Total		
		Attributes (Inputs)	Target (Outputs)	Instances
Synthetic curve	A very simple data fitting benchmark	1	1	94
Gauss3	Two blended Gaussians on decaying exponential baseline plus noise included	1	1	250
Engine behavior	Contain the behavior of two attributes, these are Torque and Nitrous oxide emissions	2	2	1199
Chemical sensor	It contains the measurements taken from eight sensors during a chemical process	8	1	498
Abalone shell rings	It contains eight attributes that describe different shells	8	1	4177
Bodyfat percentage	It contains thirteen attributes with which we can estimate the percentage of body fat of a person	13	1	252

The random sub-sampling validation technique was used, for which 30 experiments were defined, where for each experiment a new test set is constructed, distributed in the following way; 60% for training, 20% for validation and 20% for testing, in order to avoid overfitting and overtraining, and to ensure the robustness of the neuro-fuzzy system used.

One of the critical training hyperparameters, which help to minimize the error function during training, if it is established with the appropriate value, is the number of rules, to be able to choose the right value to the evaluated dataset. Per dataset 30 experiments were done, a range of minimum and maximum values were established that could be assigned as a rule number value, for each value of the range, training was executed and the error obtained was stored, finally, that rule number value associated with a minor error result was counted and the one that had more frequency was the value chosen as adequate, to perform the complete experimentation and obtain the general results.

The metrics on which the comparative analysis was based were *R* correlation coefficient, *R*^2^ coefficient of determination and *RMSE* root mean square error. We start by evaluating and analyzing the results obtained from all the datasets used for the first metric. As we can see in Table 2 those learning methods that obtained the best means and maximum results, as well as those that presented greater stability by showing lower standard deviation, have been highlighted in bold.

**Table 2:** Results obtained with the evaluation of the R metric.

Dataset	Learning methods	Min	Mean	Max	Std	% Stability in Experimentation	Rules	Epochs
Synthetic curve	Batch	0.9144	**0.9703**	**0.9985**	**0.0239**	53	**5**	417
	Online	0.8770	0.9477	0.9935	0.0257	**77**	9	315
	Mini-Batch	0.9044	0.9698	0.9975	0.0259	70	15	**148**
Gauss3	Batch	0.9957	**0.9975**	**0.9983**	**0.0007**	77	**6**	360
	Online	0.9705	0.9791	0.9952	0.0062	40	**6**	**178**
	Mini-Batch	0.9725	0.9953	0.9968	0.0043	**97**	7	296
Bodyfat percentage	Batch	0.2060	0.6696	0.8401	0.1407	**93**	**6**	217
	Online	0.0403	0.5093	0.8405	0.1879	80	14	535
	Mini-Batch	0.7031	**0.8217**	**0.8560**	**0.0325**	53	15	**99**
Chemical sensor	Batch	-0.8198	0.4900	0.8200	0.5850	83	**6**	91
	Online	-0.8272	0.6126	0.8339	**0.4672**	**90**	16	**23**
	Mini-Batch	-0.8182	**0.6407**	**0.8358**	0.4760	**90**	**6**	37
Engine behavior (output 1)	Batch	0.8017	0.9383	0.9658	0.0317	**90**	15	544
	Online	0.9296	0.9673	0.9895	**0.0140**	73	15	**178**
	Mini-Batch	0.9403	**0.9702**	**0.9946**	0.0153	80	**10**	721
Engine behavior (output 2)	Batch	0.7273	**0.8656**	**0.8994**	0.0331	**93**	15	544
	Online	0.7633	0.8634	0.9376	0.0382	73	15	**178**
	Mini-Batch	0.8459	0.8919	0.9652	**0.0254**	77	**10**	721
Abalone shell rings	Batch	0.5460	0.5956	0.6852	0.0358	50	**10**	943
	Online	0.5397	0.5480	0.5541	**0.0034**	50	12	**34**
	Mini-Batch	0.5862	**0.6025**	**0.6246**	0.0069	**57**	16	147

**Fig 4:**
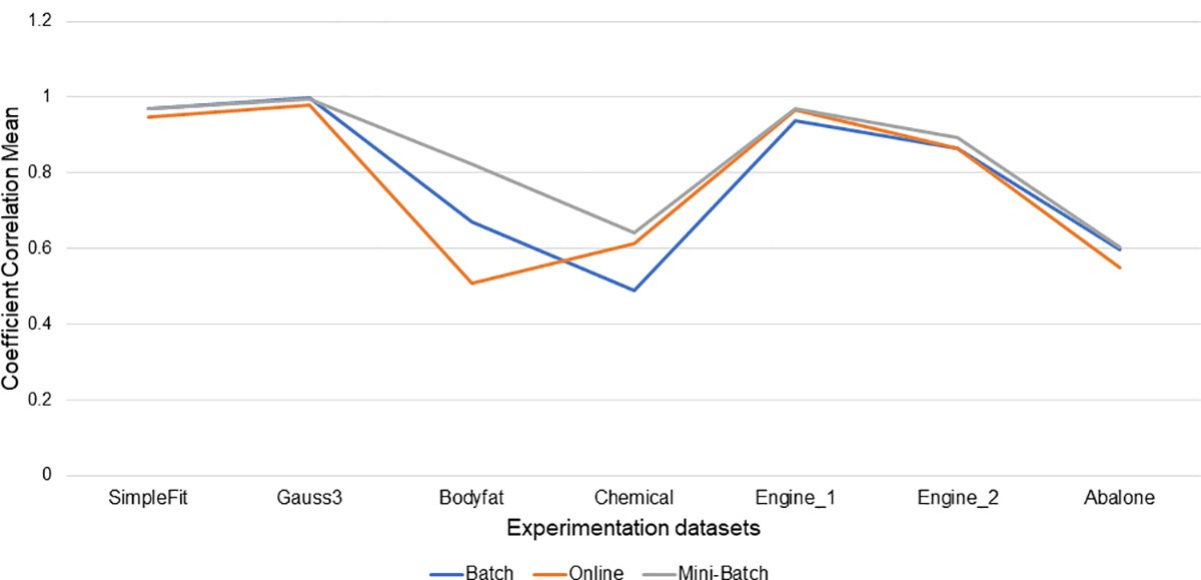
Best mean results of R by learning method.

As can be observed in Table 2, the highest means of correlation coefficients ($\bar{R}$) compared between learning methods and processed dataset were for; mini-batch with 5 best $\bar{R}$ from a total of 7 processed datasets, with a representation percentage of 71% of its experiments with the best $\bar{R}$ obtained, followed by full batch with 2 better $\bar{R}$ from a total of 7 processed datasets, showing a percentage representing 29% of their experiments with better $\bar{R}$ obtained, in no case the mean result of the online learning method was better than mini-batch and full batch, however, as can be observed in Fig 4, their differences with respect to the full batch and mini-batch learning methods were not very large.

**Fig 5:**
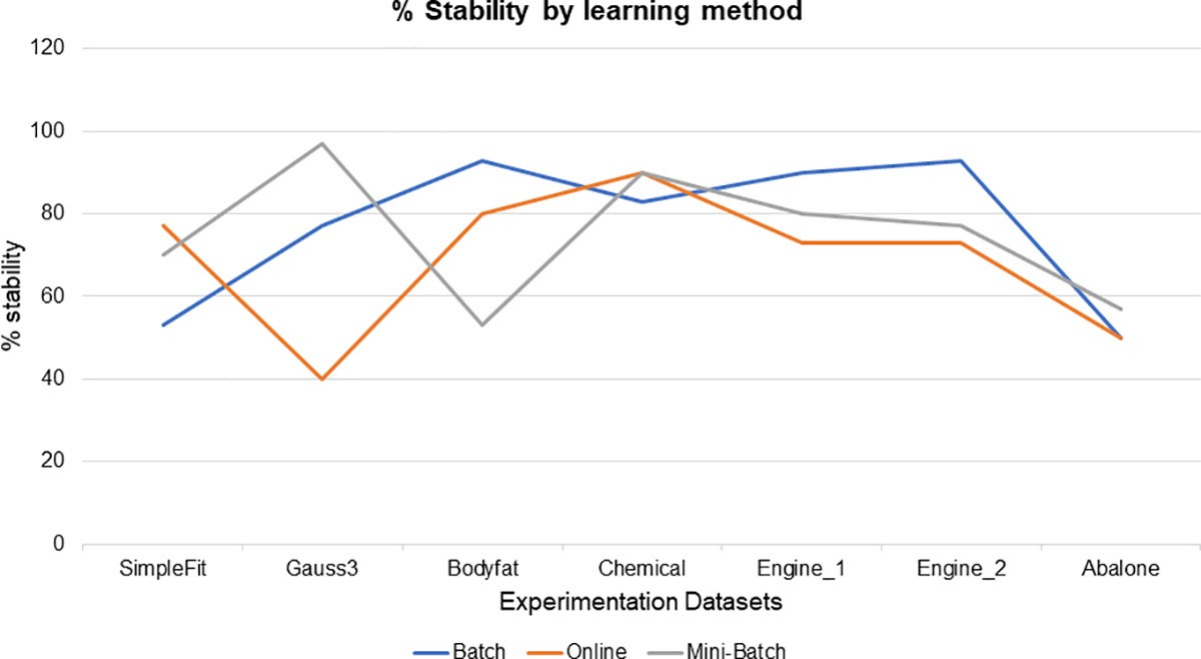
Stability among learning methods.

An indicator was defined as % stability, this indicator was calculated as follows, for each learning method, dataset processed and experiment executed, its R results that went from the mean to higher were counted (remember that the experimentation consists of 30 runs), this allows monitoring the stability of the neuro-fuzzy system. Mini-batch learning method, was which obtained 4 of 7 datasets processed with a stability of 57%, followed by the full batch learning method was which obtained 3 out of 7 data sets processed with a 43% stability, finally, the online learning method got 29% stability, it should be noted that in the chemical data set both the mini-batch and online learning method obtained the same result, as can be seen in Fig 5, the differences between stability percentages each learning method are not very large, only for gauss3 dataset case with online learning method, and the bodyfat dataset with the mini-batch learning method, some minimal peaks are observed with respect to those that obtained a greater percentage of stability, outside theses, the rest of datasets and their % stability are shown with very close differences.

**Fig 6:**
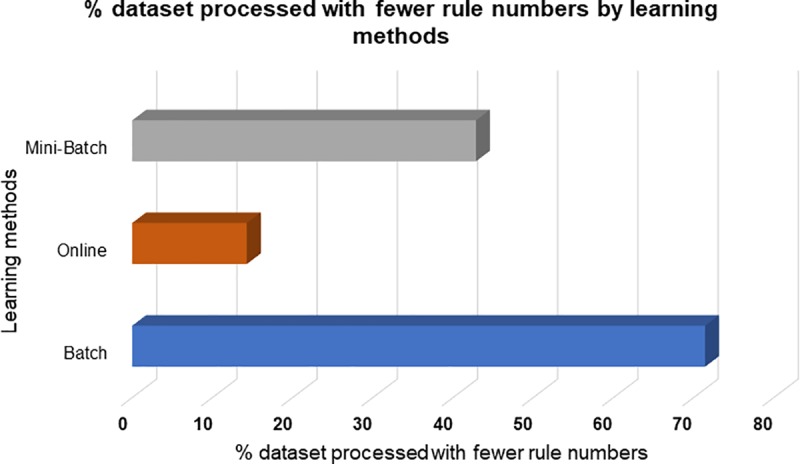
Comparative of rules number by learning method.

Another important indicator is the rules number since this hyperparameter refers to the capacity of the model to generalize a solution surface, consequently, it is considered that the smaller the rules number, the greater will be their ability to have a generalized model. The results that were obtained were the following; 71% of the datasets that were processed under the full batch learning method were processed with fewer rules, that is, 5 of 7 datasets, followed by the mini-batch learning method with 43% representing 3 of 7 processed datasets and finally the online learning method which obtained 14% with only 1 of 7 processed datasets, as it is shown in Fig 6.

**Fig 7:**
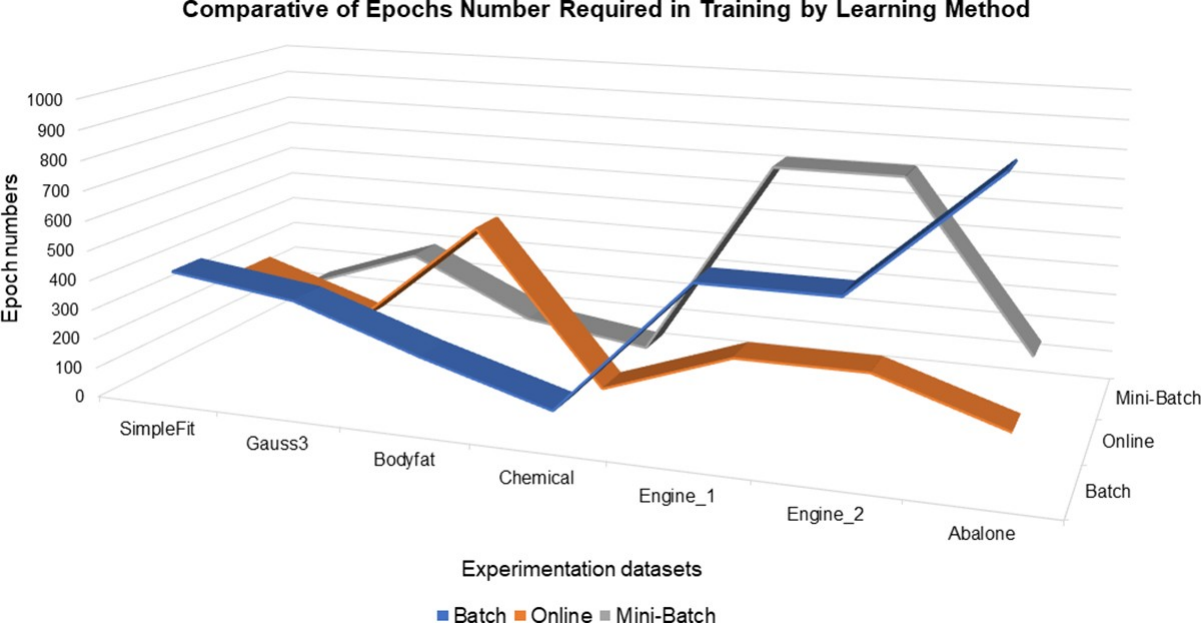
Comparative of epochs number required in training by learning method.

Finally, an indicator that allows observing neuro-fuzzy system performance with respect to the computational load that could be represented by the iterations number necessary to the processing of the dataset under different learning methods, it is known as the epochs number required during the training stage to generate the model. As it is shown in Fig 7, who required more epochs number were the Full Batch and Mini-Batch learning methods, of the which the latter in 2 of 7 processed datasets trained in fewer numbers of times than the rest of the learning methods with a representation percentage of 29%, at all times the full batch learning method exceeded the epochs number required for training, and who generated their training in fewer epochs number was the online learning method with 5 of 7 datasets processed and a representation percentage of 71% with a lower epochs number required in training, however, the fact that it has ended early, it does not mean that it has achieved a better correlation coefficient or a better convergence, as shown in the previous figures, really, the online learning method behavior obeyed the validation criterion that monitored if the error function no longer decreased then the training was interrupted.

**Fig 8:**
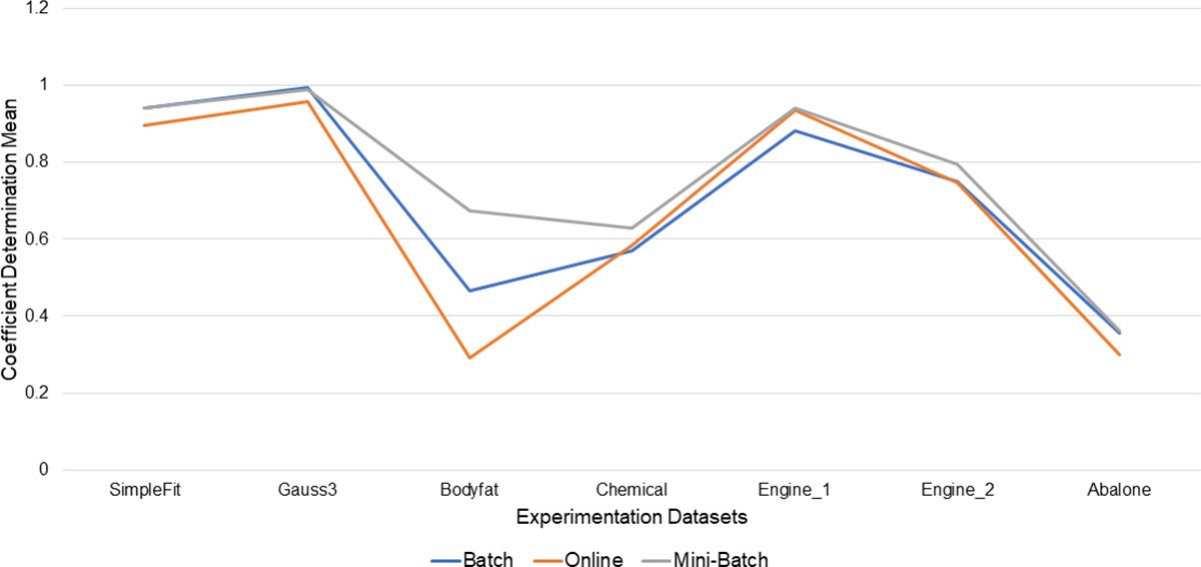
Best mean results of R^2^ by learning method.

Another metric analyzed was the coefficient of determination *R*^2^, which allows us to know how well the generated model fits the data, as shown in Table 3, the results that were obtained from the Mini-Batch learning method was 71% of the best *R*^2^ with 5 of 7 processed datasets, followed by the Full Batch learning method with 2 of 7 datasets processed with the best mean results of *R*^2^ with a percentage of representation of 29%, and even though the online learning method did not obtain the best means in any of the processed datasets with respect to the other learning methods, their differences were not as significant. These frequencies are shown in Fig 8.

**Table 3:** Results obtained with the evaluation of the R^2^ metric.

Dataset	Learning methods	Min	Mean	Max	Std	% Stability in Experimentation
Synthetic curve	Batch	0.9144	**0.9415**	**0.9970**	**0.0467**	50
	Online	0.7666	0.8977	0.9869	0.0488	**70**
	Mini-Batch	0.8160	0.9406	0.9950	0.0503	**70**
Gauss3	Batch	0.9914	**0.9950**	**0.9967**	**0.0013**	77
	Online	0.9417	0.9585	0.9904	0.0123	40
	Mini-Batch	0.9456	0.9906	0.9936	0.0085	**97**
Bodyfat percentage	Batch	0.0386	0.4654	0.7045	0.1543	83
	Online	-0.0024	0.2907	0.7053	0.1761	80
	Mini-Batch	0.4923	**0.6749**	**0.7317**	**0.0519**	**93**
Chemical sensor	Batch	0.0639	0.5701	0.6718	0.1731	83
	Online	0.2577	0.5854	0.6948	**0.1097**	80
	Mini-Batch	0.1403	**0.6288**	**0.6980**	0.1152	**87**
Engine behavior (output 1)	Batch	0.6424	0.8813	0.9327	0.0565	**90**
	Online	0.8641	0.9357	0.9791	**0.0269**	87
	Mini-Batch	0.8841	**0.9415**	**0.9893**	0.0297	80
Engine behavior (output 2)	Batch	0.5285	0.7502	0.8087	0.0539	**93**
	Online	0.5823	0.7467	0.8790	0.0651	90
	Mini-Batch	0.7152	**0.7960**	**0.9316**	**0.0458**	73
Abalone shell rings	Batch	0.2980	0.3558	**0.4694**	0.0436	50
	Online	0.2911	0.3001	0.3069	**0.0037**	50
	Mini-Batch	0.3435	**0.3628**	0.3900	0.0084	**57**

**Fig 9:**
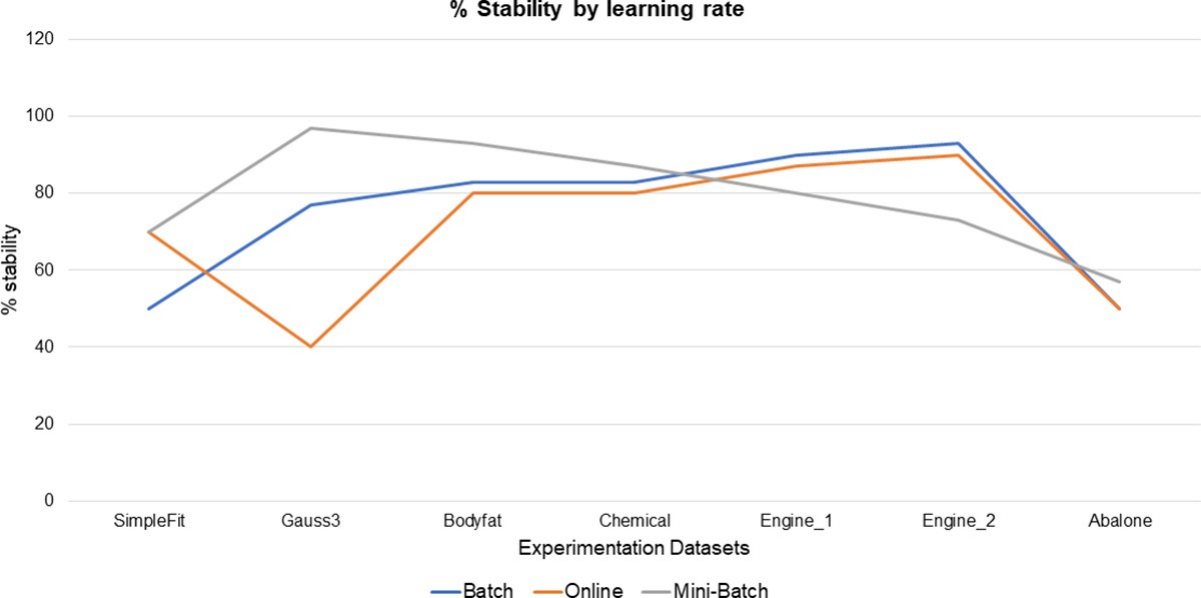
% Stability among learning methods with respect to R^2^ metric.

For the purposes of the indicator % stability by learning methods, as shown in Fig 9, it is considered as stable the experiments that obtained R^2^ results from the mean upwards, so for each dataset processed and for each experiment executed in said dataset, the experiments that obtained results from mean or bigger were counted, based on this, it was obtained that for the Mini Batch learning method its stability percentage was 71% (5 of 7 datasets processed), followed by the full batch learning method with 29% stability (2 of 7 datasets processed) and finally the online learning method with 14% stability (with 1 of 7 processed dataset).

**Fig 10:**
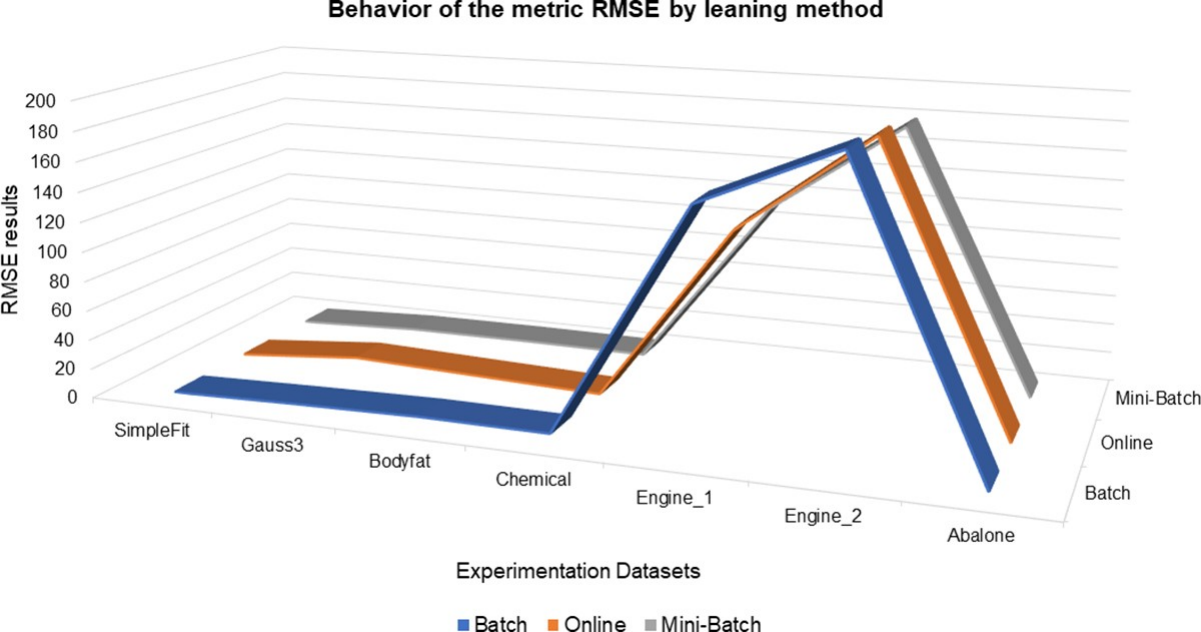
Behavior of the metric RMSE by learning method.

Finally, the last metric evaluated is the root mean square error RMSE, which shows the differences obtained between the estimated and expected model, as shown in Table 4 and Fig 10, the behavior and trends among the three learning methods is very similar, however, the learning method that achieved a greater decrease in error based on the RMSE metric (Root Mean Square Error) was mini-batch with 4 of 7 datasets processed, with a percentage of representation of 57%, as well as for full batch and online learning methods with 2 of 7 processed datasets and 29% representation percentage.

**Table 4:** Results obtained with the evaluation of the RMSE metric.

Dataset	Learning methods	Min	Mean	Max	Std	C.V.
Synthetic curve	Batch	0.16	**0.60**	1.10	0.30	49.11
	Online	0.30	0.83	1.29	**0.20**	**24.55**
	Mini-Batch	0.20	**0.60**	1.05	0.26	44.28
Gauss3	Batch	2.31	**2.81**	3.66	**0.35**	**12.34**
	Online	3.89	7.90	9.59	1.40	17.75
	Mini-Batch	3.20	3.72	9.06	1.02	27.38
Bodyfat percentage	Batch	3.68	4.55	5.57	0.46	10.09
	Online	3.53	4.83	6.13	0.60	12.34
	Mini-Batch	3.30	**3.84**	**4.58**	**0.33**	**8.59**
Chemical sensor	Batch	2.47	4.12	8.48	1.17	**28.45**
	Online	0.24	**1.86**	**3.58**	**0.85**	45.96
	Mini-Batch	0.44	3.19	7.55	1.35	42.43
Engine behavior (output 1)	Batch	130.08	157.58	200.51	**15.19**	**9.64**
	Online	77.71	121.56	180.56	23.91	19.67
	Mini-Batch	55.80	**116.75**	**159.06**	28.72	24.60
Engine behavior (output 2)	Batch	174.42	196.19	282.54	22.84	11.64
	Online	127.60	188.21	235.54	22.94	12.19
	Mini-Batch	113.88	**178.34**	**203.48**	**19.51**	**10.94**
Abalone shell rings	Batch	1.14	1.43	1.57	0.11	7.86
	Online	0.21	**0.35**	**0.56**	0.07	20.91
	Mini-Batch	1.40	1.48	1.60	**0.04**	**2.85**

Hereunder, we will analyze obtained results from the perspective of applied learning methods, to try to highlight some relevant characteristics of each of the evaluated learning method, Table 5 allows us to compare the efficiency and performance based on the mean results of chosen metrics.

**Table 5:** Comparative table of mean results obtained from the experiments executed with all the datasets used.

Learning Methods	$\bar{R}$	$C.V.=f(\bar{R})$	$\bar{R^{2}}$	$C.V.=f(\bar{R^{2}})$	% stability in experimentation	Rules	Epoch
Batch	0.7708	19.42	0.6906	14.61	82	8	429
Online	0.7520	20	0.6456	15.51	76	12	211
Mini-Batch	0.8268	14.12	0.7444	6.50	81	9	241

**Fig 11:**
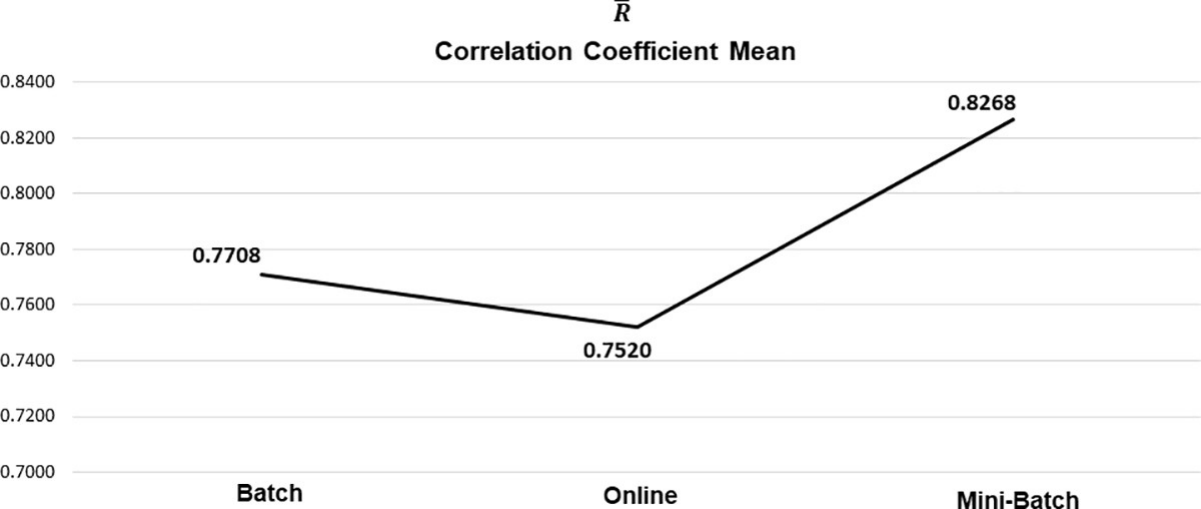
Correlation coefficient mean by learning method.

**Fig 12:**
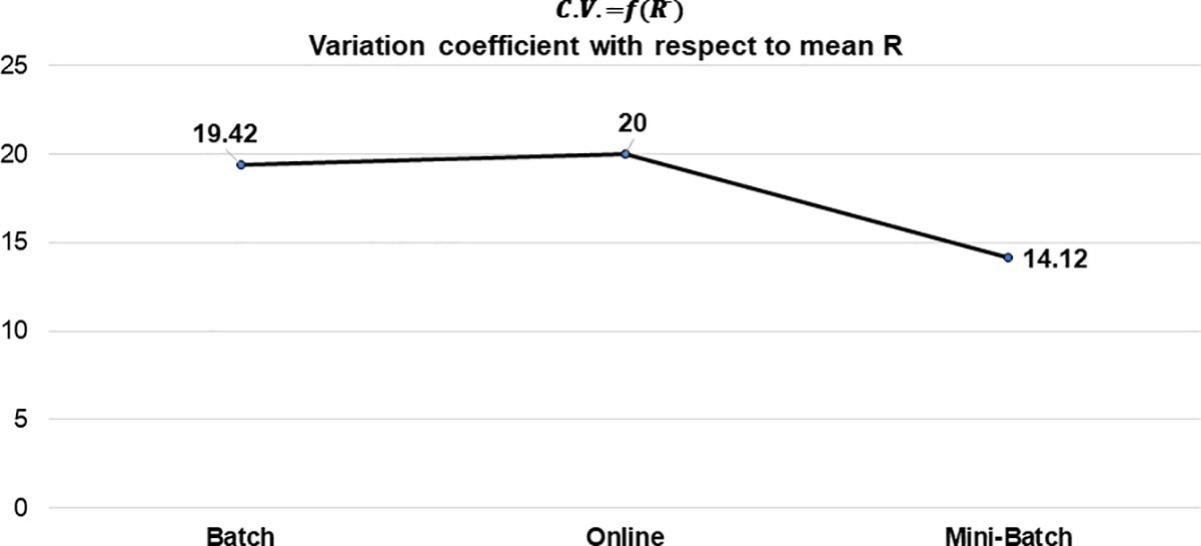
Variation coefficient with respect to mean R by learning method.

As previously mentioned, the composition of these results is based on the mean obtained from all the experiments generated from the different datasets analyzed, for each applied learning method. As seen in Table 5, the general tendency of $\bar{R}$ was positive, with the mini-batch learning method showing the greatest strength in its correlation and better control in its variability, this can be clearly seen in Fig 11 and Fig 12 respectively.

**Fig 13:**
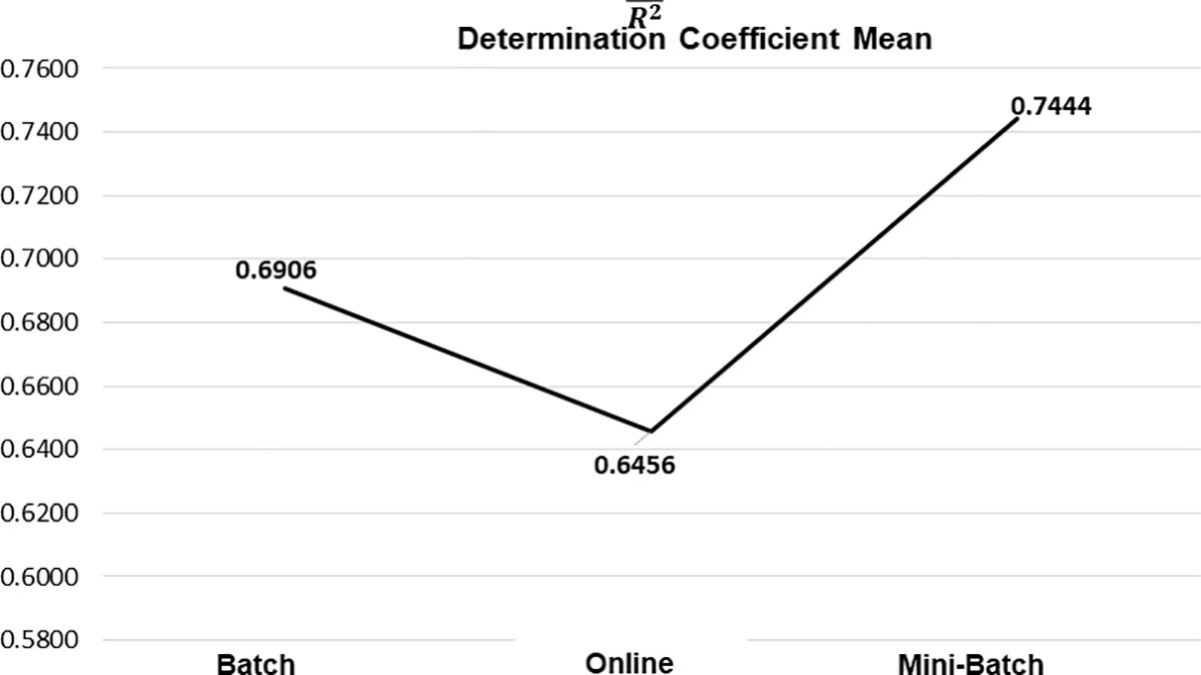
Determination coefficient mean by each learning method.

**Fig 14:**
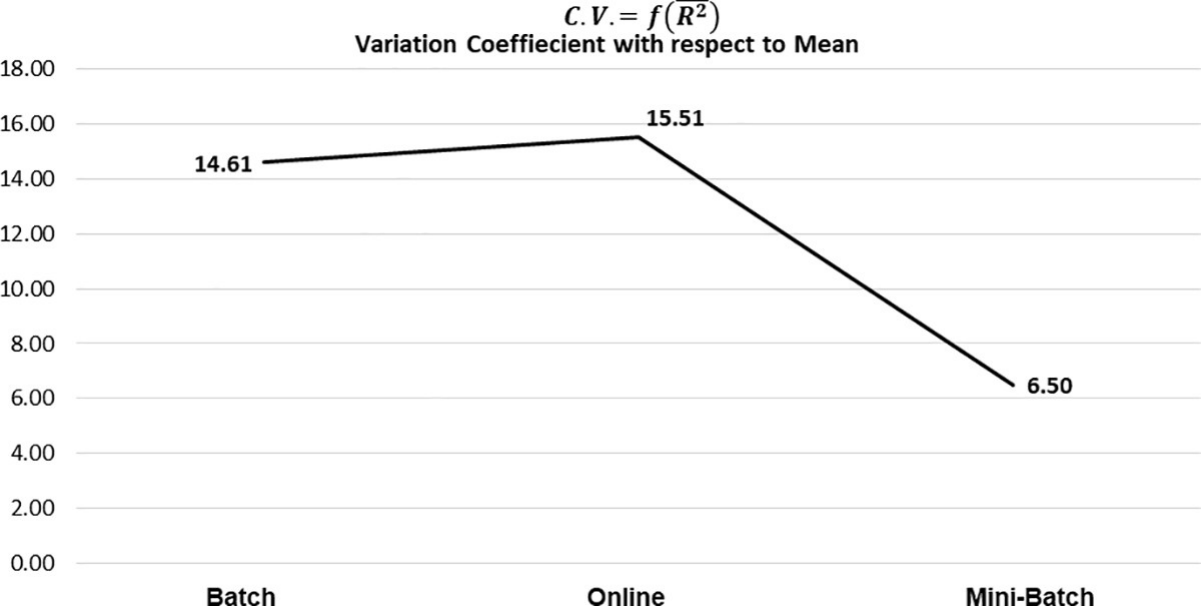
Variation coefficient mean by each learning method.

The models obtained from the learning methods based on mini-batch were better adjusted to the data, so they presented a better explanation of the variability of the estimated values with respect to the expected ones, as presented in Fig 13 and Fig 14 respectively.

**Fig 15:**
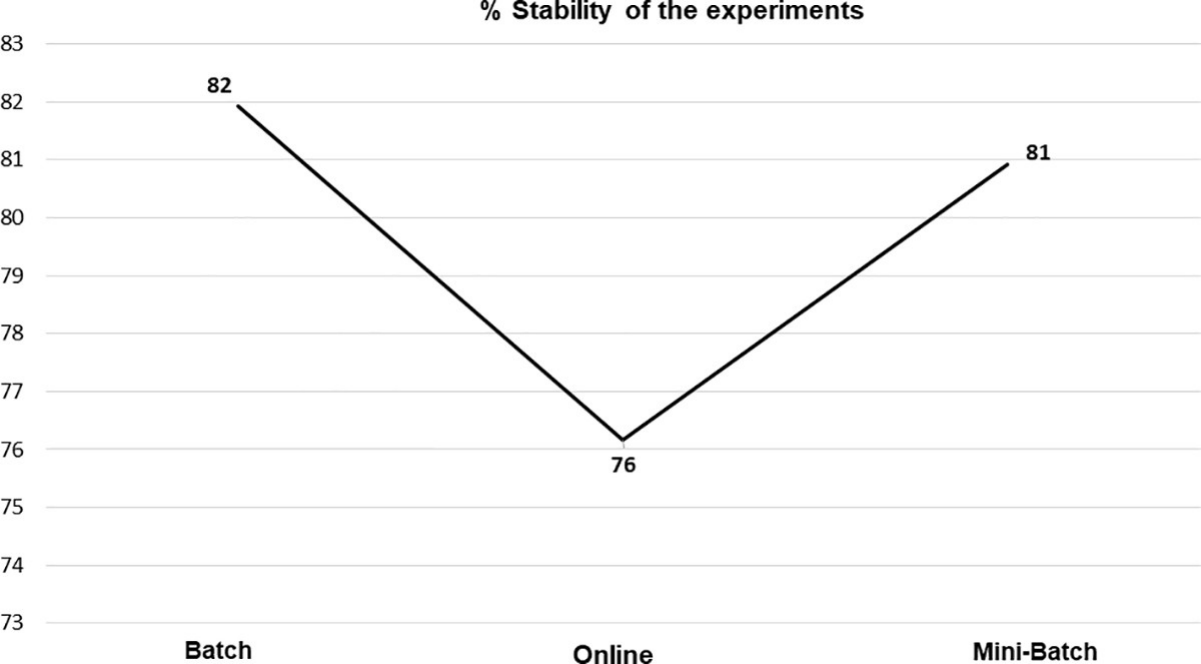
% stability of the experiments by each learning method.

Stability studies were carried out, that is, from each experiment processed, how many obtained correlation coefficients from the mean to the maximum, with the batch learning method obtaining the highest percentage, but only by one percentage point with respect to mini-batch, as can be seen, both in Table 5 and Fig 15.

**Fig 16:**
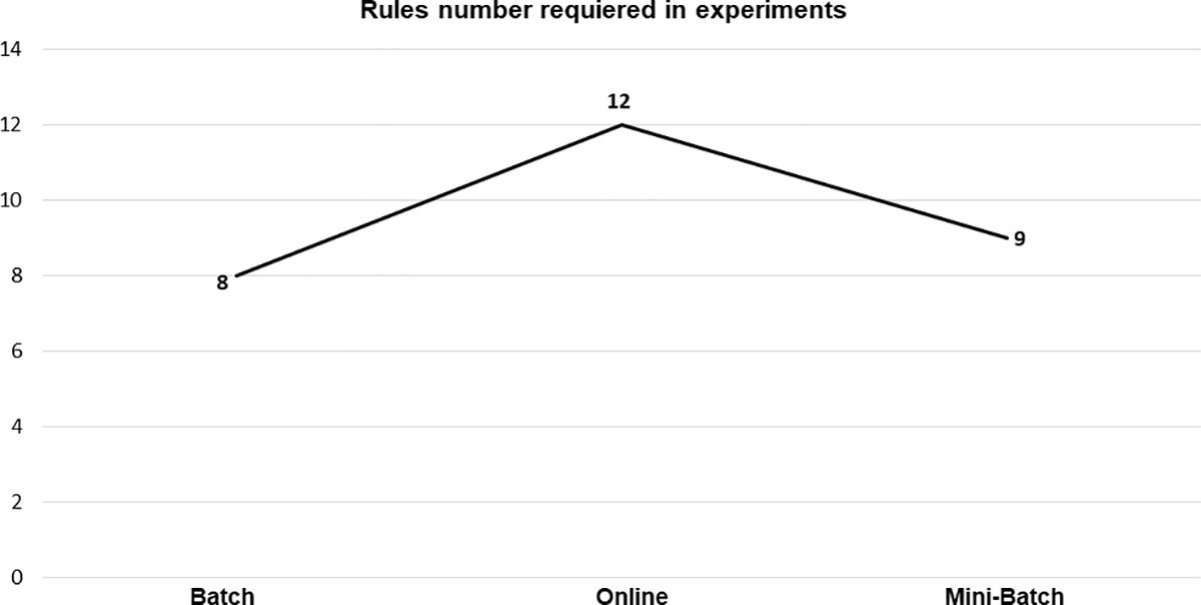
Rules experiments by each learning method.

The rules number mean required for the training shows that both the batch and mini-batch learning methods required fewer rules number during their experimentation than the online-based learning, as can be seen in Fig 16, however, it was this the last one who finished his training in fewer epochs number, this being an advantage with respect to batch, because as shown previously both have very close results in the metrics of $\bar{R}$ and $\bar{R^{2}}$, however, the stability of the online learning was below both batch learning and mini-batch.

In order to evaluate the goodness of fit of the trained model with the neuro-fuzzy system proposed under the different learning methods, an error minimization performance analysis was executed using the following metrics; SSE (Sum of Squared Error), MSE (Mean-Squared Error) and RMSE (Root Mean-Squared Error), generated in two moments, the first one occurred in training where for each dataset (training, validation and tests) results were obtained from aforementioned metrics and finally after the model was adjusted, the test is generated with the complete entries to evaluate its goodness of fit, also under the same metrics.

According to Table 6 and Table 7, it is observed that 3 out of 6 datasets used in the experimentation with respect to its mean value calculated had greater error reduction, representing 50% of the processed datasets, with the mini-batch learning method being the one had better control in the minimization of the error and that also showed more stability as can be observed in the results obtained in the standard deviation.

**Table 6:** Comparative analysis about the behavior of goodness of fit based on SSE, MSE and RMSE per learning method and dataset processed (part 1).

Dataset	Learning Method	Stages where metrics were calculated	SSE				MSE				RMSE			
			Min	Mean	Max	Std	Min	Mean	Max	Std	Min	Mean	Max	Std
**Synthetic curve**	**Batch**	**Training**	1.23E+00	1.04E+02	2.30E+03	3.44E+02	2.94E-03	2.50E-01	5.52E+00	8.26E-01	0.05	**0.27**	2.35	0.42
		**Validation**	6.80E-01	3.72E+01	8.69E+02	1.29E+02	1.63E-03	8.93E-02	2.08E+00	3.09E-01	0.04	**0.16**	1.44	0.25
		**Test**	3.77E-01	2.65E+01	5.74E+02	8.55E+01	9.04E-04	6.35E-02	1.38E+00	2.05E-01	0.03	**0.13**	1.17	0.22
		**Test in adjusted model**	2.28E+00	4.11E+01	1.11E+02	3.21E+01	2.47E-02	4.46E-01	1.20E+00	3.49E-01	0.16	0.60	1.10	0.30
	**Online**	**Training**	1.80E+00	**3.49E+01**	2.02E+02	**3.32E+01**	5.69E-03	**1.10E-01**	6.38E-01	**1.05E-01**	0.08	0.29	0.80	**0.17**
		**Validation**	8.22E+00	**2.35E+01**	5.94E+02	**3.49E+01**	2.60E-02	**7.43E-02**	1.88E+00	**1.10E-01**	0.16	0.25	1.37	**0.10**
		**Test**	5.51E+00	**1.99E+01**	1.28E+02	**1.49E+01**	1.74E-02	**6.29E-02**	4.06E-01	**4.72E-02**	0.13	0.24	0.64	**0.09**
		**Test in adjusted model**	8.52E+00	6.63E+01	1.52E+02	3.01E+01	9.26E-02	7.21E-01	1.65E+00	3.28E-01	0.30	0.83	1.29	**0.20**
	**Mini-Batch**	**Training**	1.95E+00	8.42E+01	2.20E+03	2.93E+02	1.32E-02	5.69E-01	1.48E+01	1.98E+00	0.11	0.49	3.85	0.57
		**Validation**	1.22E+00	3.13E+01	8.38E+02	1.19E+02	8.26E-03	2.12E-01	5.66E+00	8.04E-01	0.09	0.27	2.38	0.37
		**Test**	9.19E-01	2.32E+01	7.31E+02	9.33E+01	6.21E-03	1.57E-01	4.94E+00	6.30E-01	0.08	0.24	2.22	0.32
		**Test in adjusted model**	3.77E+00	**3.88E+01**	1.02E+02	**3.00E+01**	4.10E-02	**4.22E-01**	1.11E+00	**3.27E-01**	0.20	**0.60**	1.05	**0.26**
**Gauss 3**	**Batch**	**Training**	7.91E+02	1.40E+04	2.14E+05	**2.67E+04**	2.20E+00	3.89E+01	5.94E+02	**7.42E+01**	1.48	4.18	24.37	4.63
		**Validation**	2.28E+02	4.12E+03	2.81E+05	**1.60E+04**	6.34E-01	1.15E+01	7.81E+02	**4.46E+01**	0.80	2.26	27.94	2.52
		**Test**	2.76E+02	5.10E+03	8.02E+04	**9.78E+03**	7.67E-01	1.42E+01	2.23E+02	**2.72E+01**	0.88	2.50	14.92	2.82
		**Test in adjusted model**	1.33E+03	**1.98E+03**	3.33E+03	**5.07E+02**	5.35E+00	**7.99E+00**	1.34E+01	**2.04E+00**	2.31	**2.81**	3.66	**0.35**
	**Online**	**Training**	2.68E+03	1.51E+05	7.54E+05	2.36E+05	1.51E+01	8.46E+02	4.23E+03	1.32E+03	3.88	20.05	65.07	21.13
		**Validation**	7.44E+02	5.35E+04	2.70E+05	8.43E+04	4.18E+00	3.01E+02	1.52E+03	4.74E+02	2.05	11.91	38.96	12.64
		**Test**	1.02E+03	5.68E+04	2.88E+05	8.96E+04	5.73E+00	3.19E+02	1.62E+03	5.03E+02	2.39	12.41	40.25	12.90
		**Test in adjusted model**	3.76E+03	1.60E+04	2.28E+04	4.62E+03	1.52E+01	6.43E+01	9.20E+01	1.86E+01	3.89	7.90	9.59	1.40
	**Mini-Batch**	**Training**	1.36E+03	**7.08E+03**	7.33E+05	4.89E+04	4.59E+00	**2.39E+01**	2.47E+03	1.65E+02	2.14	**3.00**	49.75	**3.87**
		**Validation**	7.60E+02	**2.88E+03**	2.81E+05	2.03E+04	2.57E+00	**9.72E+00**	9.50E+02	6.85E+01	1.60	**1.98**	30.82	**2.41**
		**Test**	4.17E+02	**1.75E+03**	1.75E+05	1.12E+04	1.41E+00	**5.90E+00**	5.90E+02	3.79E+01	1.19	**1.62**	24.30	**1.81**
		**Test in adjusted model**	2.55E+03	3.69E+03	2.04E+04	3.16E+03	1.03E+01	1.49E+01	8.22E+01	1.28E+01	3.20	3.72	9.06	1.02
**Bodyfat percentage**	**Batch**	**Training**	2.39E+03	7.09E+03	5.67E+04	6.15E+03	1.10E+01	3.27E+01	2.61E+02	2.84E+01	3.32	5.42	16.16	1.82
		**Validation**	1.10E+03	1.99E+03	2.13E+04	2.13E+03	5.09E+00	9.17E+00	9.80E+01	**9.81E+00**	2.26	**2.87**	9.90	**0.95**
		**Test**	1.24E+03	2.29E+03	1.56E+04	1.55E+03	5.72E+00	1.05E+01	7.19E+01	7.14E+00	2.39	**3.15**	8.48	0.78
		**Test in adjusted model**	3.39E+03	5.23E+03	7.74E+03	1.04E+03	1.36E+01	2.09E+01	3.10E+01	4.17E+00	3.68	4.55	5.57	0.46
	**Online**	**Training**	2.82E+03	1.01E+04	6.40E+04	1.41E+04	8.00E+00	**2.87E+01**	1.81E+02	4.00E+01	2.83	**4.66**	13.46	2.65
		**Validation**	2.82E+03	1.01E+04	6.40E+04	1.41E+04	8.00E+00	2.87E+01	1.81E+02	4.00E+01	2.83	4.66	13.46	2.65
		**Test**	1.33E+03	3.81E+03	2.12E+04	4.60E+03	3.76E+00	1.08E+01	6.01E+01	1.30E+01	1.94	2.96	7.75	1.43
		**Test in adjusted model**	3.11E+03	5.93E+03	9.39E+03	1.47E+03	1.24E+01	2.37E+01	3.76E+01	5.89E+00	3.53	4.83	6.13	0.60
	**Mini-Batch**	**Training**	3.00E+03	**4.27E+03**	2.84E+04	**2.75E+03**	3.03E+01	4.31E+01	2.87E+02	**2.78E+01**	5.51	6.43	16.94	**1.35**
		**Validation**	8.76E+02	**1.30E+03**	2.20E+04	**2.19E+03**	8.85E+00	**1.31E+01**	2.22E+02	**2.21E+01**	2.97	3.38	14.91	1.32
		**Test**	8.51E+02	**1.06E+03**	4.81E+03	**4.36E+02**	8.60E+00	**1.07E+01**	4.86E+01	**4.40E+00**	2.93	3.24	6.97	**0.48**
		**Test in adjusted model**	2.73E+03	**3.71E+03**	5.24E+03	**6.39E+02**	1.09E+01	**1.49E+01**	2.10E+01	**2.56E+00**	3.30	**3.84**	4.58	**0.33**

**Table 7:** Comparative analysis about the behavior of goodness of fit based on SSE, MSE and RMSE per learning method and dataset processed (part 2).

Dataset	Learning Method	Stages where metrics were calculated	SSE				MSE				RMSE			
			Min	Mean	Max	Std	Min	Mean	Max	Std	Min	Mean	Max	Std
**Chemical sensor**	**Batch**	**Training**	4.58E+03	9.67E+05	4.40E+07	5.37E+06	5.04E+01	1.06E+04	4.84E+05	5.91E+04	7.10	41.68	695.44	94.80
		**Validation**	1.94E+03	**6.08E+05**	2.55E+07	**3.21E+06**	2.13E+01	**6.68E+03**	2.81E+05	**3.53E+04**	4.62	29.86	529.80	**76.50**
		**Test**	2.07E+03	3.32E+05	1.54E+07	1.86E+06	2.28E+01	3.65E+03	1.69E+05	2.04E+04	4.77	24.62	411.54	55.44
		**Test in adjusted model**	3.03E+03	9.08E+03	3.56E+04	6.10E+03	6.11E+00	1.83E+01	7.19E+01	1.23E+01	2.47	4.12	8.48	1.17
	**Online**	**Training**	4.15E+03	**3.12E+04**	6.25E+05	**1.29E+05**	2.28E+01	**4.77E+02**	2.72E+04	**2.83E+03**	4.77	**13.47**	164.82	**17.29**
		**Validation**	1.97E+03	1.11E+06	2.56E+07	5.34E+06	2.28E+01	1.24E+04	1.11E+06	1.17E+05	4.77	**22.23**	1055.00	109.66
		**Test**	1.97E+03	**2.17E+03**	3.03E+03	**2.06E+02**	2.28E+01	**1.59E+02**	7.06E+02	**1.86E+02**	4.77	**10.85**	26.58	**6.42**
		**Test in adjusted model**	2.79E+01	**2.06E+03**	6.37E+03	**1.53E+03**	5.63E-02	**4.16E+00**	1.28E+01	**3.08E+00**	0.24	**1.86**	3.58	**0.85**
	**Mini-Batch**	**Training**	4.53E+03	1.48E+06	5.08E+07	8.35E+06	1.22E+02	4.00E+04	1.37E+06	2.26E+05	11.06	58.50	1171.93	193.81
		**Validation**	1.36E+03	8.09E+05	2.54E+07	4.21E+06	3.67E+01	2.19E+04	6.86E+05	1.14E+05	6.06	41.03	828.48	144.05
		**Test**	1.75E+03	1.29E+05	4.35E+06	7.14E+05	4.73E+01	3.47E+03	1.18E+05	1.93E+04	6.88	20.93	343.04	55.86
		**Test in adjusted model**	9.62E+01	5.92E+03	2.82E+04	5.09E+03	1.94E-01	1.19E+01	5.69E+01	1.03E+01	0.44	3.19	7.55	1.35
**Engine behavior (output 1)**	**Batch**	**Training**	4.89E+07	8.97E+07	1.36E+09	1.55E+08	8.99E+04	1.65E+05	2.50E+06	2.84E+05	299.83	368.61	1581.40	170.70
		**Validation**	1.83E+07	3.46E+07	5.27E+08	6.23E+07	3.37E+04	6.36E+04	9.69E+05	1.14E+05	183.54	226.94	984.63	109.93
		**Test**	1.95E+07	3.35E+07	4.85E+08	5.47E+07	3.59E+04	6.15E+04	8.92E+05	1.01E+05	189.49	227.34	944.27	99.39
		**Test in adjusted model**	2.03E+07	3.00E+07	4.81E+07	**6.02E+06**	1.69E+04	2.51E+04	4.02E+04	**5.03E+03**	130.08	157.58	200.51	**15.19**
	**Online**	**Training**	2.55E+07	6.60E+07	1.01E+09	1.02E+08	6.89E+03	9.82E+04	6.29E+06	3.41E+05	83.02	199.42	2508.62	241.91
		**Validation**	8.82E+06	2.37E+07	4.71E+08	4.21E+07	6.89E+03	3.96E+04	2.92E+06	1.36E+05	83.02	147.34	1710.13	133.72
		**Test**	8.07E+06	2.16E+07	1.63E+08	2.64E+07	6.89E+03	3.66E+04	1.01E+06	9.34E+04	83.02	145.54	1006.21	124.45
		**Test in adjusted model**	7.23E+06	1.83E+07	3.90E+07	7.22E+06	6.04E+03	1.53E+04	3.26E+04	6.03E+03	77.71	121.56	180.56	23.91
	**Mini-Batch**	**Training**	1.35E+07	**3.38E+07**	1.34E+09	**7.42E+07**	1.87E+04	**4.69E+04**	1.86E+06	**1.03E+05**	136.65	**190.50**	1362.95	**103.09**
		**Validation**	3.94E+06	**1.06E+07**	4.62E+08	**2.59E+07**	5.46E+03	**1.47E+04**	6.41E+05	**3.59E+04**	73.91	**104.93**	800.78	**60.65**
		**Test**	4.97E+06	**1.12E+07**	4.00E+08	**2.10E+07**	6.89E+03	**1.55E+04**	5.55E+05	**2.92E+04**	83.02	**112.17**	745.11	**54.16**
		**Test in adjusted model**	3.73E+06	**1.73E+07**	3.03E+07	7.86E+06	3.11E+03	**1.44E+04**	2.53E+04	6.57E+03	55.80	**116.75**	159.06	28.72
**Engine behavior (output 2)**	**Batch**	**Training**	4.89E+07	8.97E+07	1.36E+09	1.55E+08	8.99E+04	1.65E+05	2.50E+06	2.84E+05	299.83	368.61	1581.40	170.70
		**Validation**	1.83E+07	3.46E+07	5.27E+08	6.23E+07	3.37E+04	6.36E+04	9.69E+05	1.14E+05	183.54	226.94	984.63	109.93
		**Test**	1.95E+07	3.35E+07	4.85E+08	5.47E+07	3.59E+04	6.15E+04	8.92E+05	1.01E+05	189.49	227.34	944.27	99.39
		**Test in adjusted model**	3.64E+07	4.67E+07	9.56E+07	1.27E+07	3.04E+04	3.90E+04	7.98E+04	1.06E+04	174.42	196.19	282.54	22.84
	**Online**	**Training**	2.55E+07	6.60E+07	1.01E+09	1.02E+08	6.89E+03	9.82E+04	6.29E+06	3.41E+05	83.02	199.42	2508.62	241.91
		**Validation**	8.82E+06	2.37E+07	4.71E+08	4.21E+07	6.89E+03	3.96E+04	2.92E+06	1.36E+05	83.02	147.34	1710.13	133.72
		**Test**	8.07E+06	2.16E+07	1.63E+08	2.64E+07	6.89E+03	3.66E+04	1.01E+06	9.34E+04	83.02	145.54	1006.21	124.45
		**Test in adjusted model**	1.95E+07	4.30E+07	6.64E+07	1.00E+07	1.63E+04	3.59E+04	5.55E+04	8.37E+03	127.60	188.21	235.54	22.94
	**Mini-Batch**	**Training**	1.35E+07	**3.38E+07**	1.34E+09	**7.42E+07**	1.87E+04	**4.69E+04**	1.86E+06	**1.03E+05**	136.65	**190.50**	1362.95	**103.09**
		**Validation**	3.94E+06	**1.06E+07**	4.62E+08	**2.59E+07**	5.46E+03	**1.47E+04**	6.41E+05	**3.59E+04**	73.91	**104.93**	800.78	**60.65**
		**Test**	4.97E+06	**1.12E+07**	4.00E+08	**2.10E+07**	6.89E+03	**1.55E+04**	5.55E+05	**2.92E+04**	83.02	**112.17**	745.11	**54.16**
		**Test in adjusted model**	1.55E+07	**3.85E+07**	4.96E+07	**7.75E+06**	1.30E+04	**3.22E+04**	4.14E+04	**6.47E+03**	113.88	**178.34**	203.48	**19.51**
**Abalone shell rings**	**Batch**	**Training**	1.39E+04	**1.62E+04**	2.24E+05	9.66E+03	1.47E+01	**1.72E+01**	2.38E+02	**1.02E+01**	3.84	**4.10**	15.42	0.60
		**Validation**	4.65E+03	**5.72E+03**	9.17E+04	**4.33E+03**	4.93E+00	**6.06E+00**	9.72E+01	**4.59E+00**	2.22	**2.42**	9.86	**0.43**
		**Test**	4.56E+03	**5.36E+03**	7.77E+04	3.39E+03	4.84E+00	**5.69E+00**	8.24E+01	3.59E+00	2.20	**2.36**	9.08	0.36
		**Test in adjusted model**	5.40E+03	8.54E+03	1.02E+04	1.28E+03	1.29E+00	2.04E+00	2.45E+00	3.07E-01	1.14	1.43	1.57	0.11
	**Online**	**Training**	2.05E+04	2.09E+04	2.37E+04	**6.15E+02**	6.02E+02	6.15E+02	6.98E+02	1.81E+01	24.54	24.79	26.42	**0.36**
		**Validation**	7.73E+03	1.01E+04	8.86E+04	1.39E+04	2.27E+02	2.98E+02	2.60E+03	4.08E+02	15.07	16.17	51.04	6.16
		**Test**	8.97E+03	9.09E+03	9.39E+03	**1.02E+02**	2.64E+02	2.67E+02	2.76E+02	**3.00E+00**	16.24	16.35	16.62	**0.09**
		**Test in adjusted model**	1.80E+02	**5.26E+02**	1.29E+03	**2.28E+02**	4.32E-02	**1.26E-01**	3.10E-01	**5.46E-02**	0.21	**0.35**	0.56	0.07
	**Mini-Batch**	**Training**	1.69E+04	2.37E+04	2.73E+05	3.27E+04	1.18E+02	1.66E+02	1.91E+03	2.28E+02	10.86	12.09	43.67	4.46
		**Validation**	4.89E+03	7.17E+03	8.90E+04	1.17E+04	3.42E+01	5.01E+01	6.22E+02	8.16E+01	5.85	6.50	24.95	2.81
		**Test**	5.57E+03	7.23E+03	8.81E+04	9.65E+03	3.89E+01	5.06E+01	6.16E+02	6.75E+01	6.24	6.72	24.83	2.34
		**Test in adjusted model**	8.21E+03	9.14E+03	1.06E+04	5.27E+02	1.97E+00	2.19E+00	2.55E+00	1.26E-01	1.40	1.48	1.60	**0.04**

**Fig 17:**
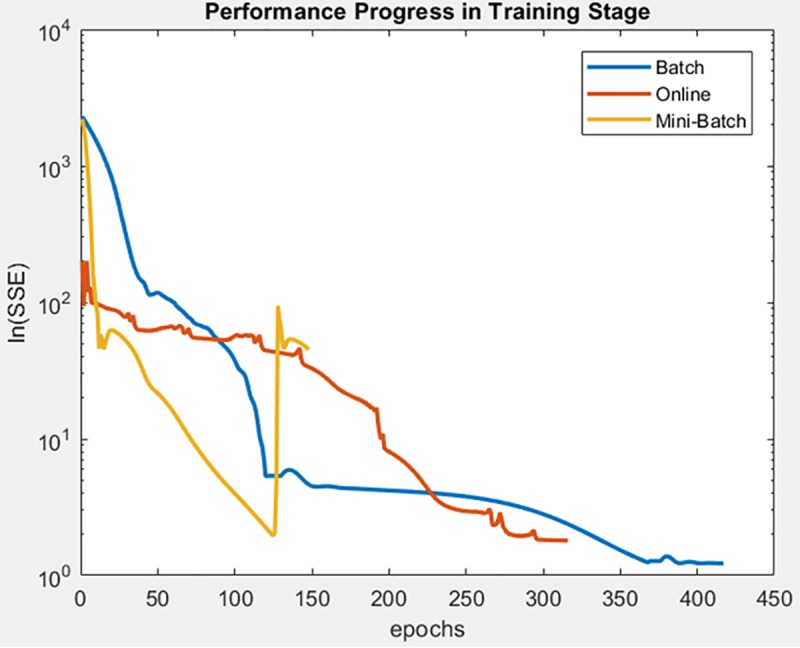
Performance progress of SSE in training stage by learning method to synthetic curve dataset.

**Fig 18:**
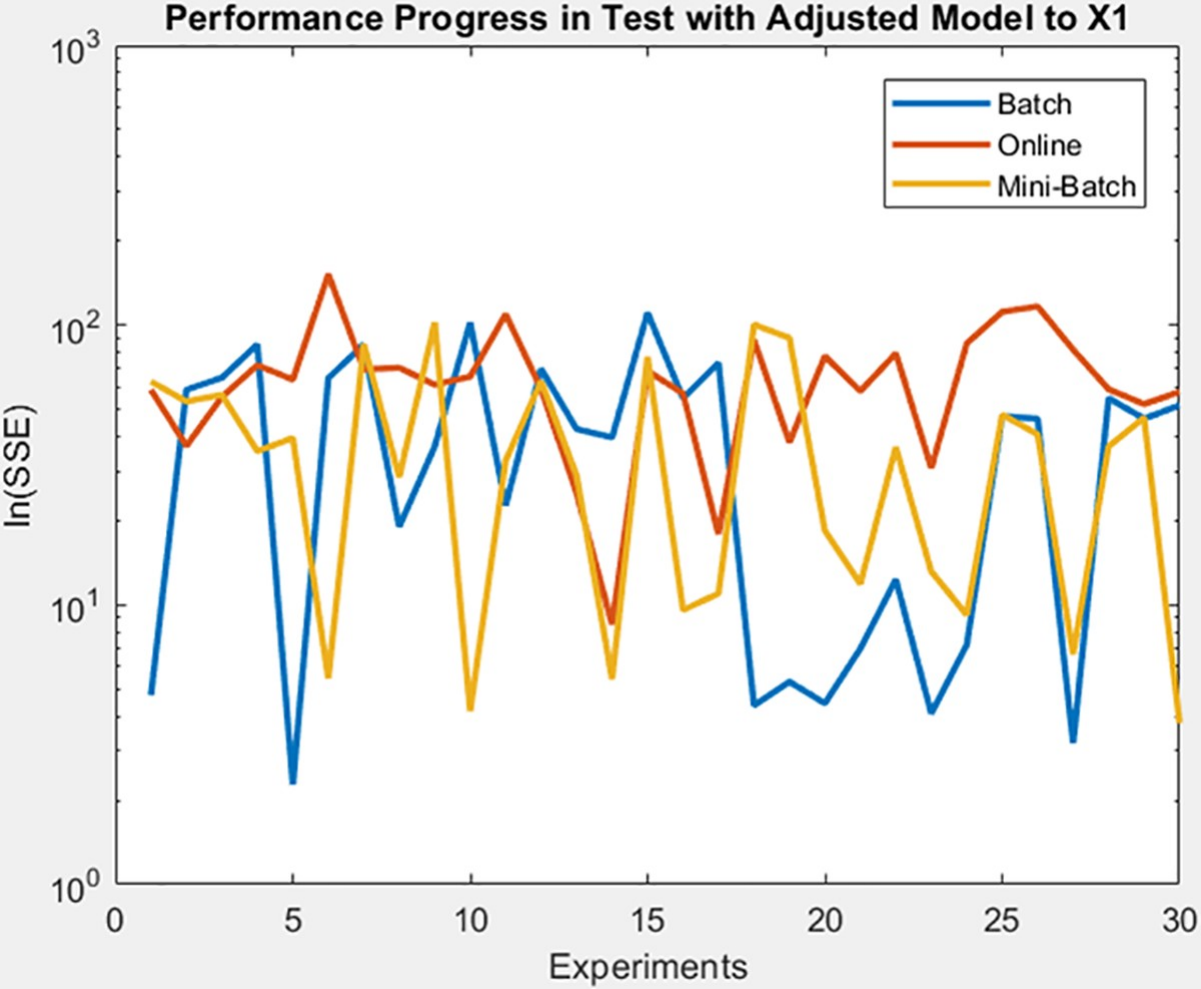
Performance progress of SSE in test stage with adjusted model by learning method to synthetic curve dataset.

Finally, the following figures show the behavior of minimization of SSE in training time and in the adjusted model test. These figures are shown below by processed dataset. The following figures correspond to synthetic curve dataset, for which in Fig 17 it is observed that the batch learning method was the one that achieved the best minimization of the SSE during training, but also in a greater epochs number. Fig 18. shows stable behavior in the minimization of the SSE, this analysis is supported in Table 6 according to its results of mean and standard deviation and it is concluded that mini-batch learning method obtained better results.

**Fig 19:**
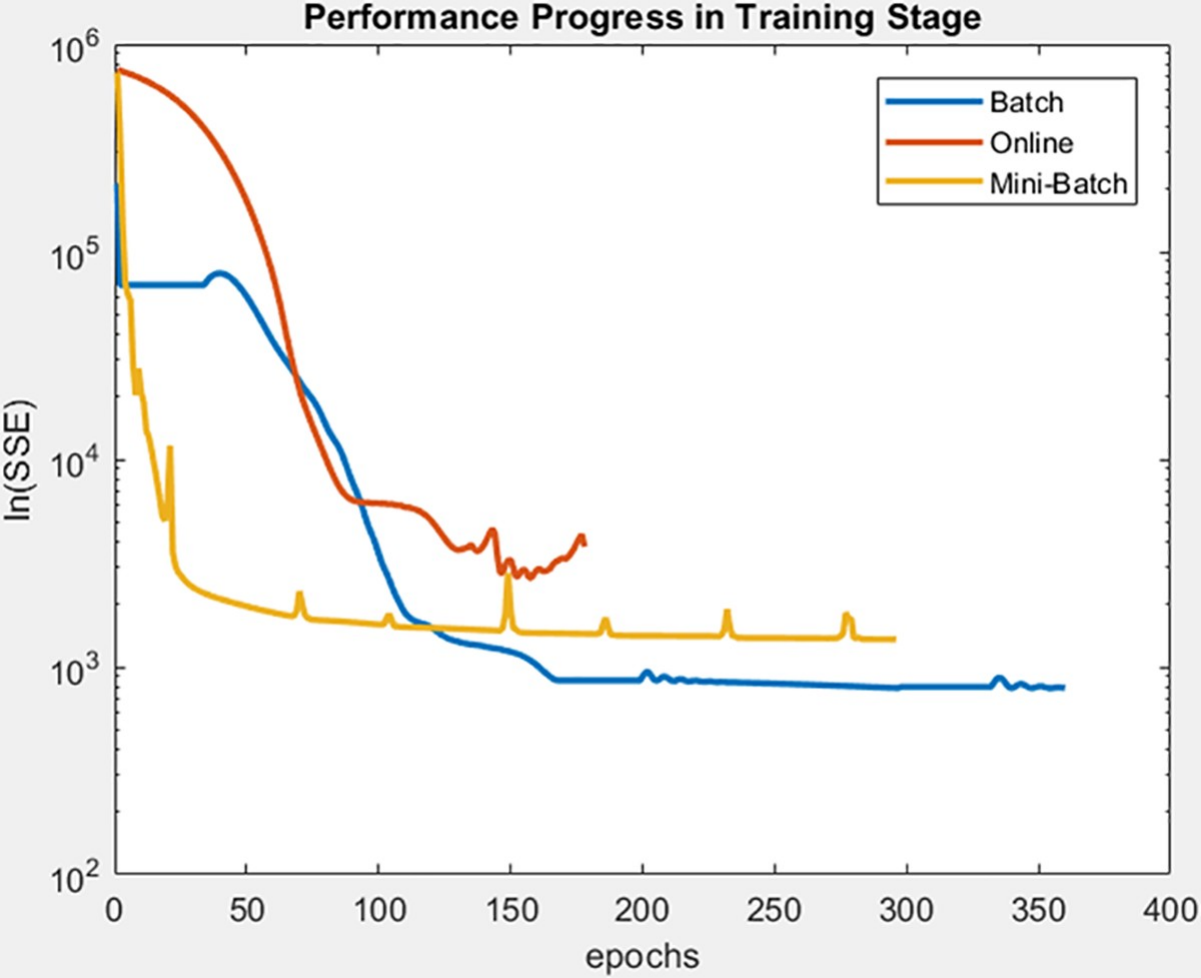
Performance progress of SEE in training stage by learning method to gauss3 dataset.

**Fig 20:**
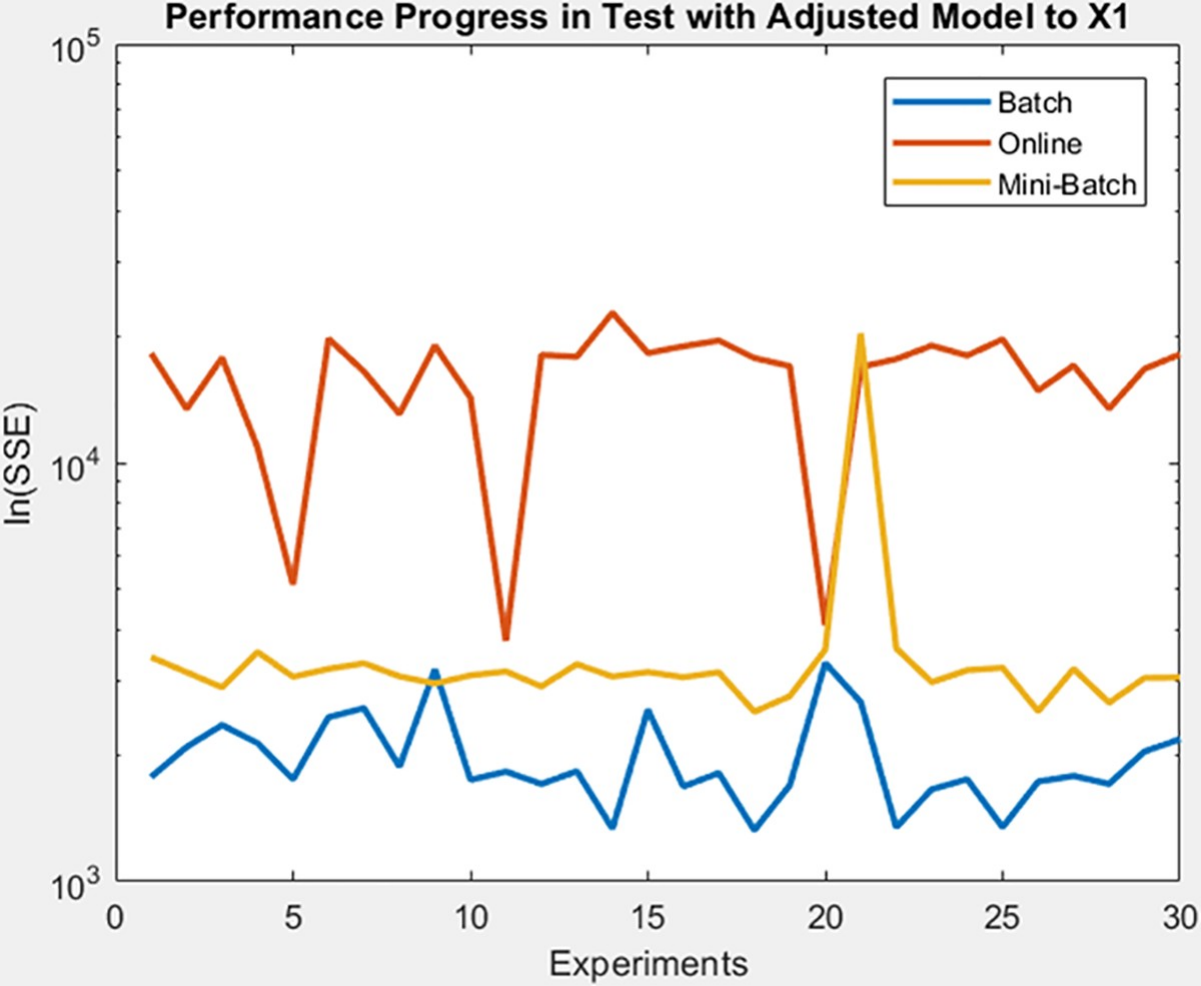
Performance progress of SEE in test stage with adjusted model by learning method to gauss3 dataset.

Continue with gauss3 dataset, for which in Fig 19 it is observed that the batch learning method was the one that achieved a better minimization of the SSE during the training, but also in a greater epochs number. It is also shown that this same learning method achieved greater stability in the minimization of the SSE when testing on the adjusted model, as can be seen in Fig 20, the analysis is supported by mean and standard deviation shown in Table 6.

**Fig 21:**
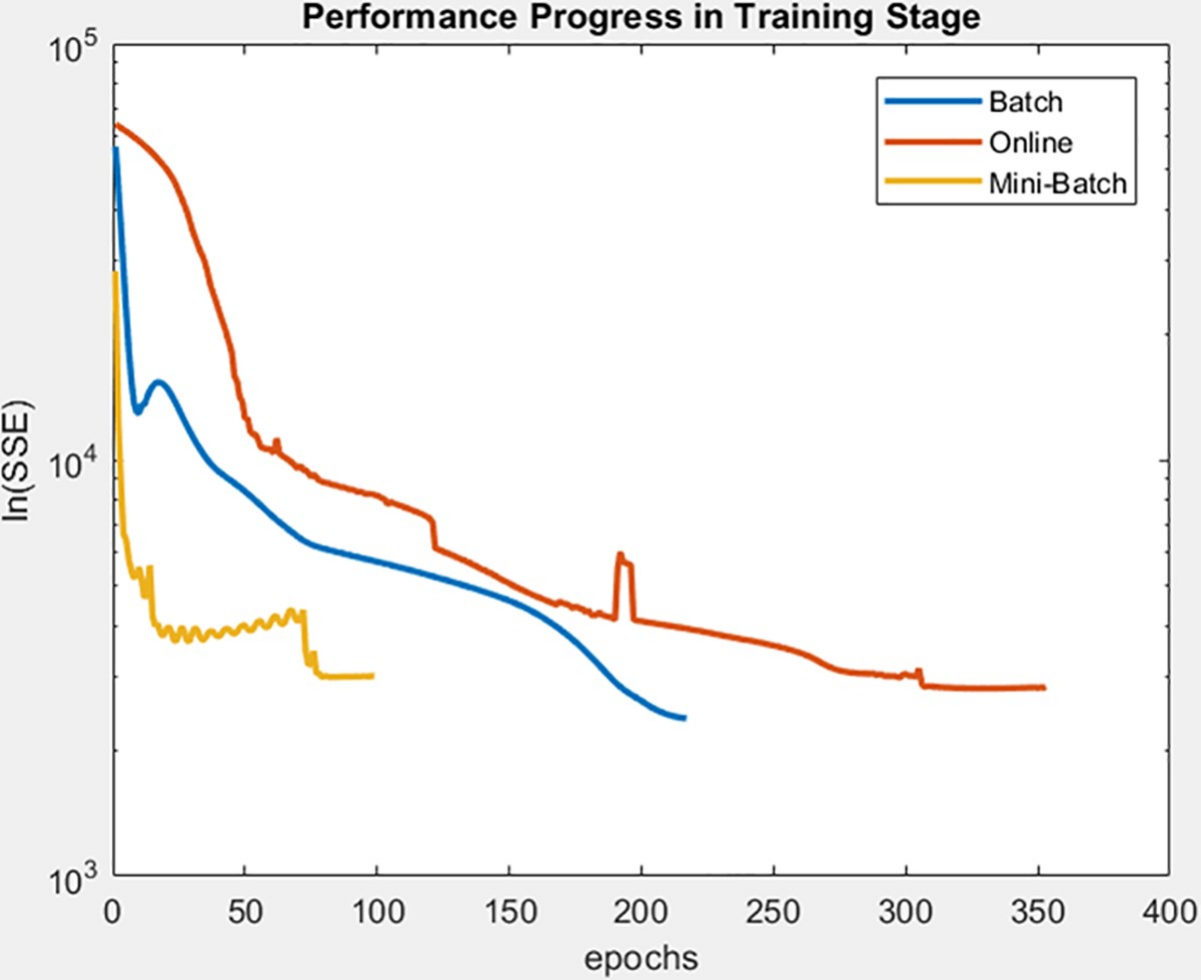
Performance progress of SEE in training stage by learning method to bodyfat percentage dataset.

**Fig 22:**
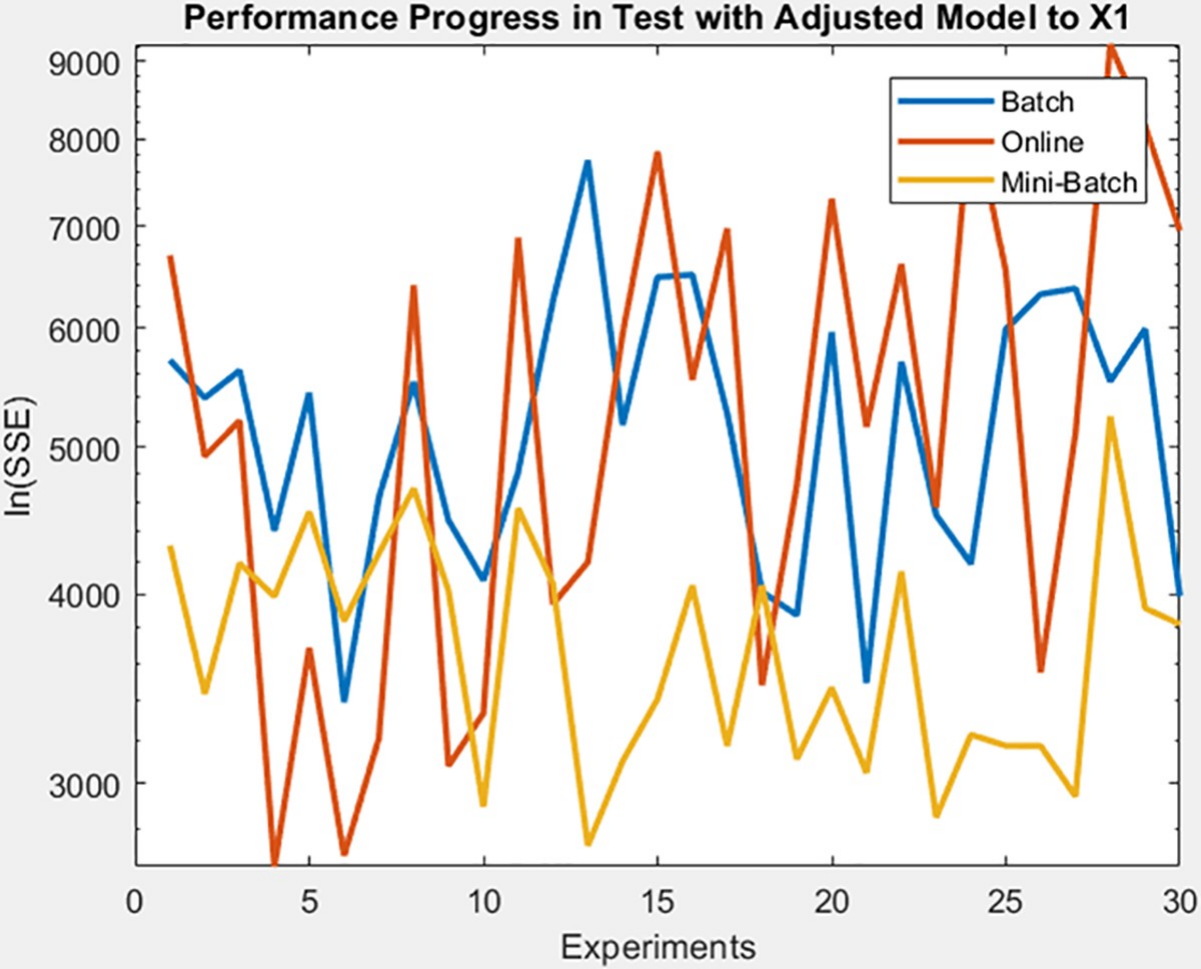
Performance progress of SEE in test stage with adjusted model by learning method to bodyfat percentage dataset.

Follows with bodyfat percentage dataset, for which in Fig 21 it is observed that the mini-batch learning method was one who achieved the best SSE minimization during training, and in a smaller epochs number. It is also shown that this same learning method achieved greater stability in the minimization of the SSE when testing on the adjusted model, as can be seen in Fig 22, also supporting the conclusion of the results through the mean and standard deviation shown in Table 6.

**Fig 23:**
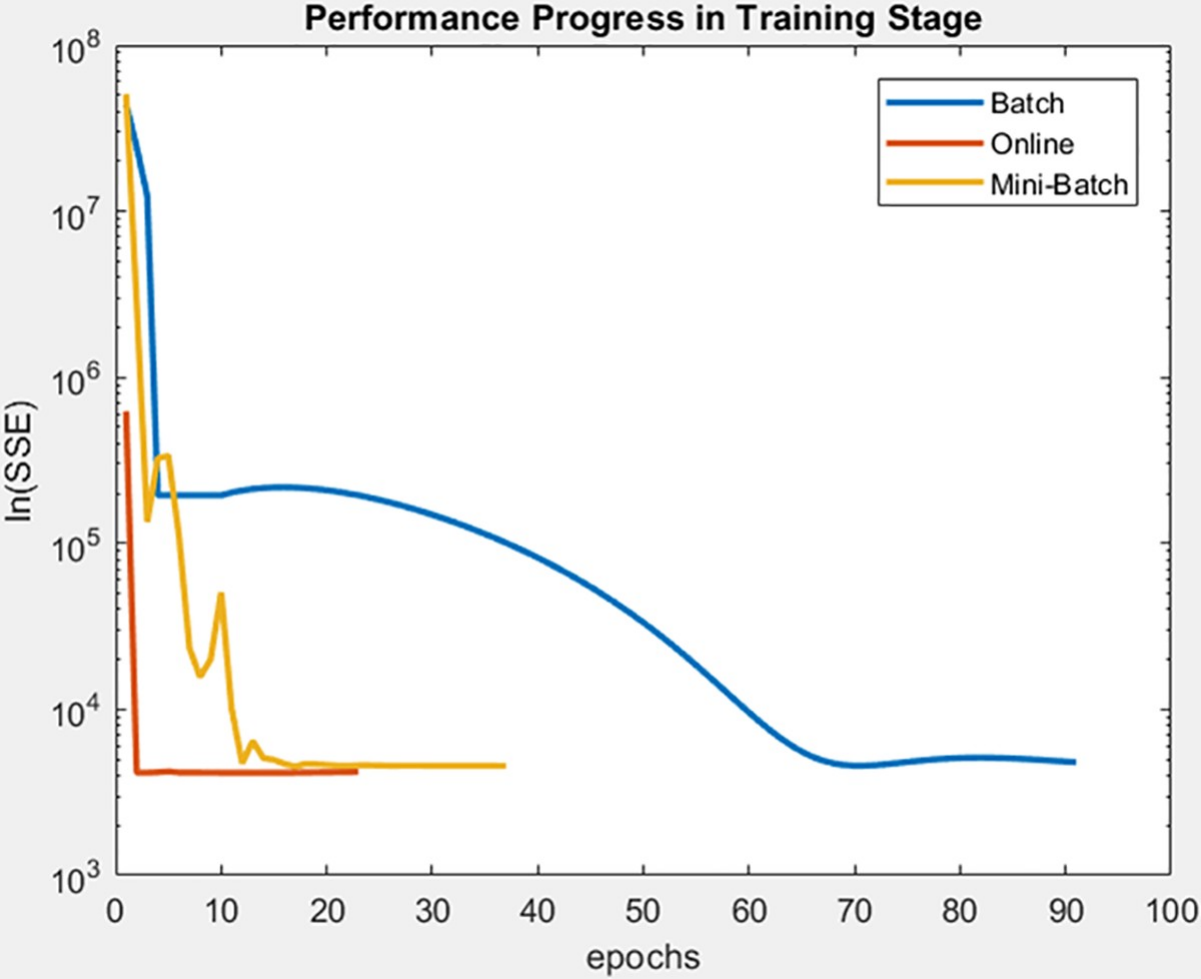
Performance progress of SEE in training stage by learning method to chemical sensor dataset.

**Fig 24:**
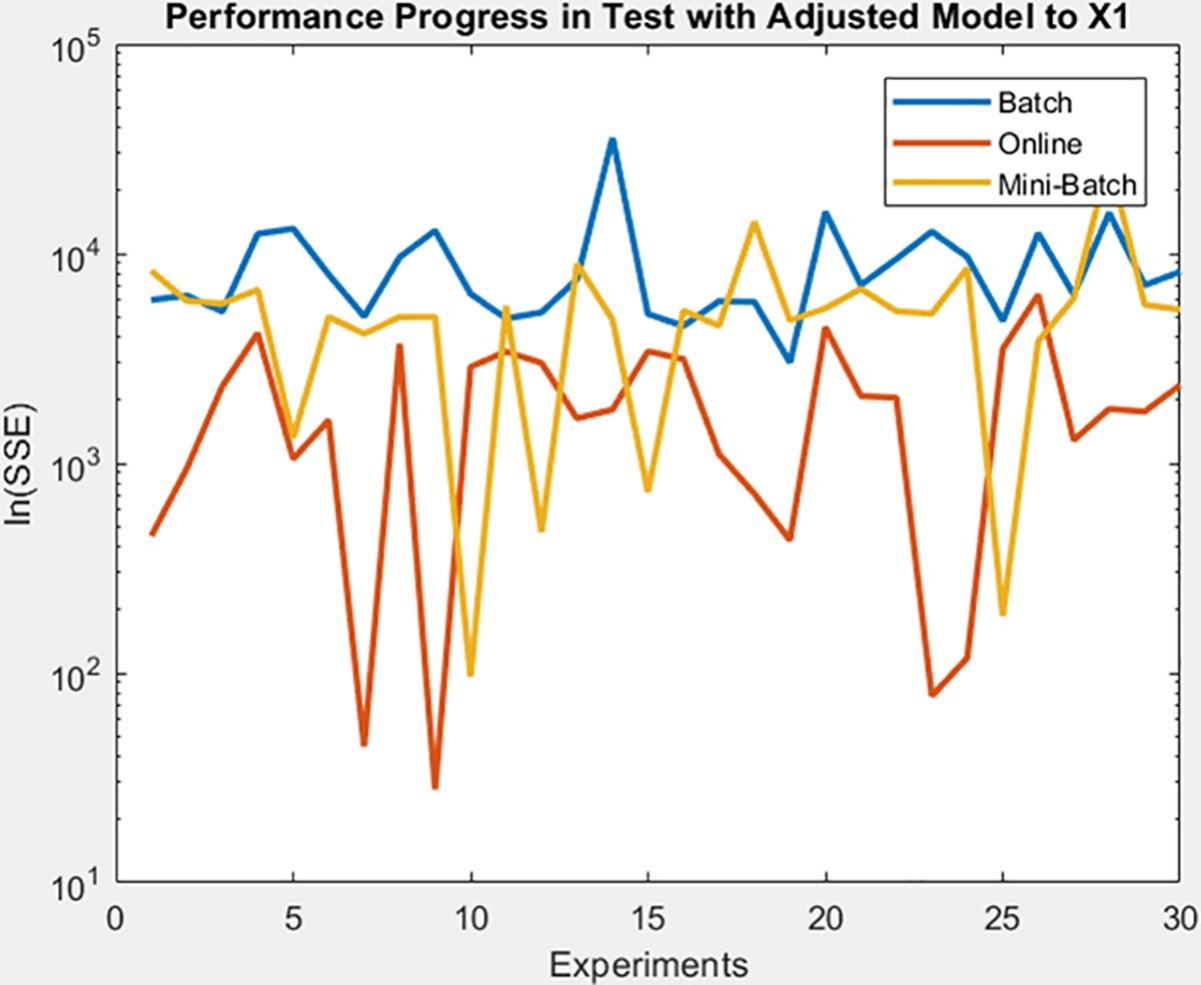
Performance progress of SEE in test stage with adjusted model by learning method to chemical sensor dataset.

Following dataset corresponds to chemical sensor dataset, for which the learning method that achieved a better minimization of the SSE both in training and in the test on the adjusted model was for online, as can be seen in Fig 23 and Fig 24 respectively, also supporting the conclusion of the mean and standard deviation results shown in Table 7.

**Fig 25:**
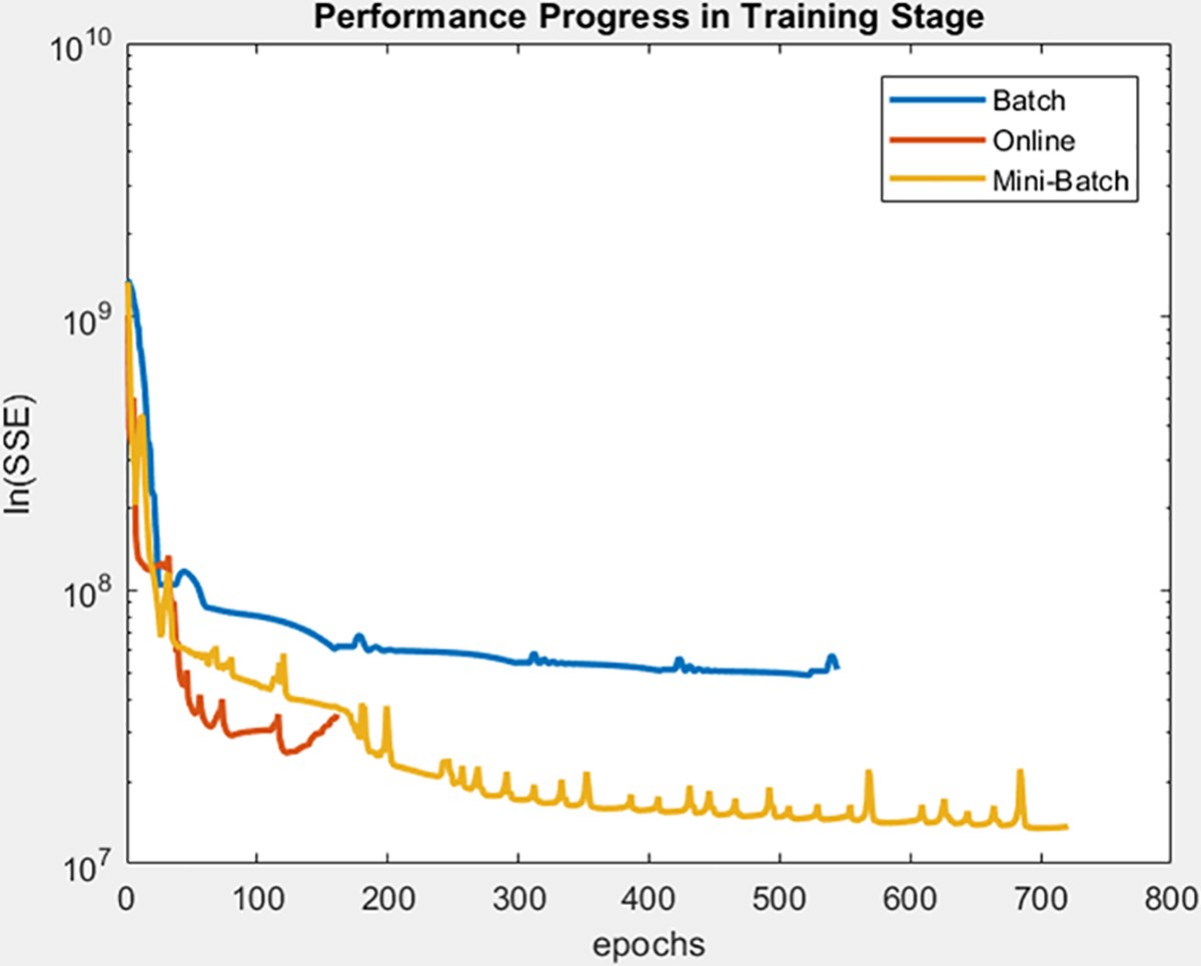
Performance progress of SEE in training stage by learning method to engine behavior dataset.

**Fig 26:**
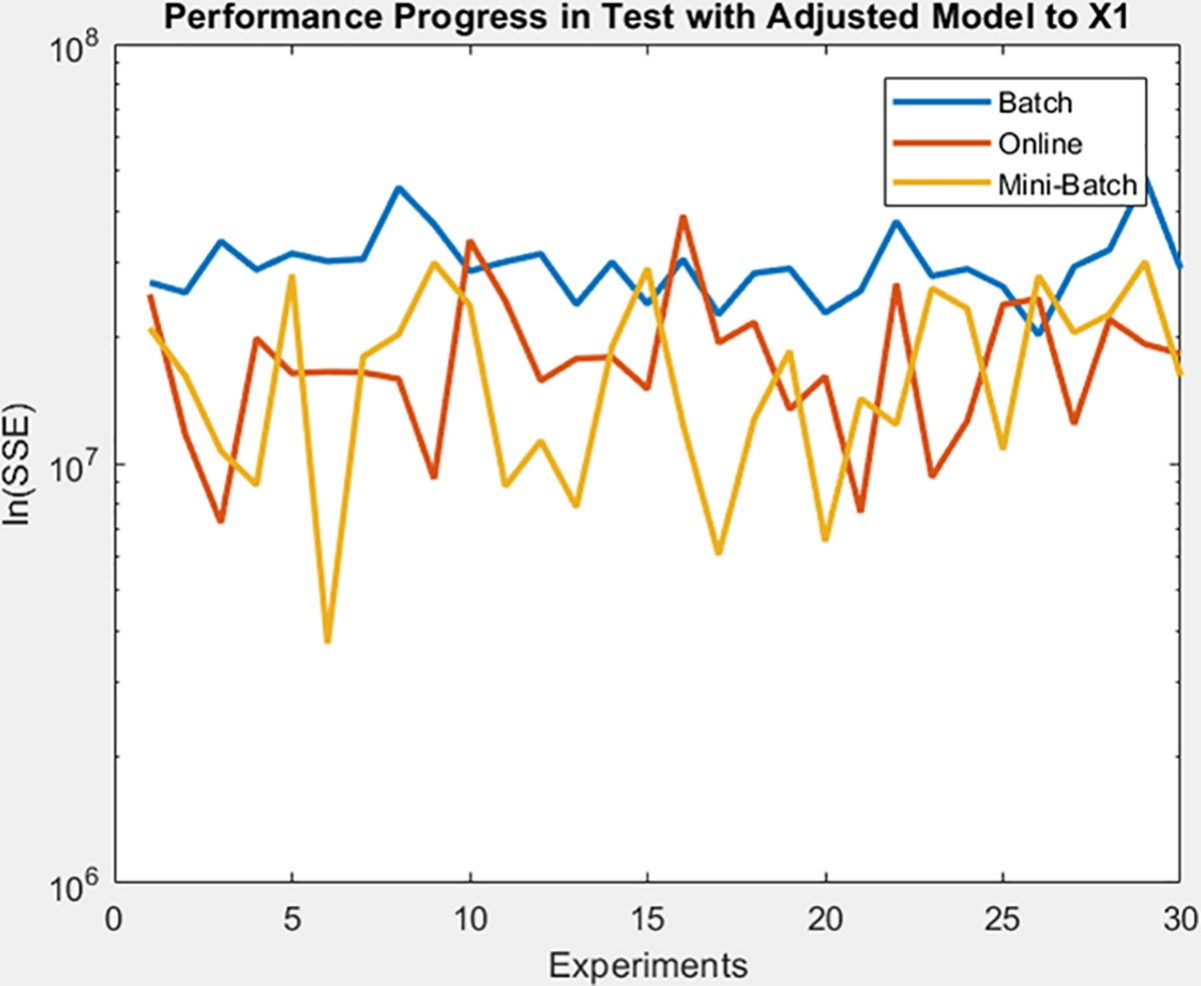
Performance progress of SEE in test stage with adjusted model by learning method to engine behavior dataset to output 1.

**Fig 27:**
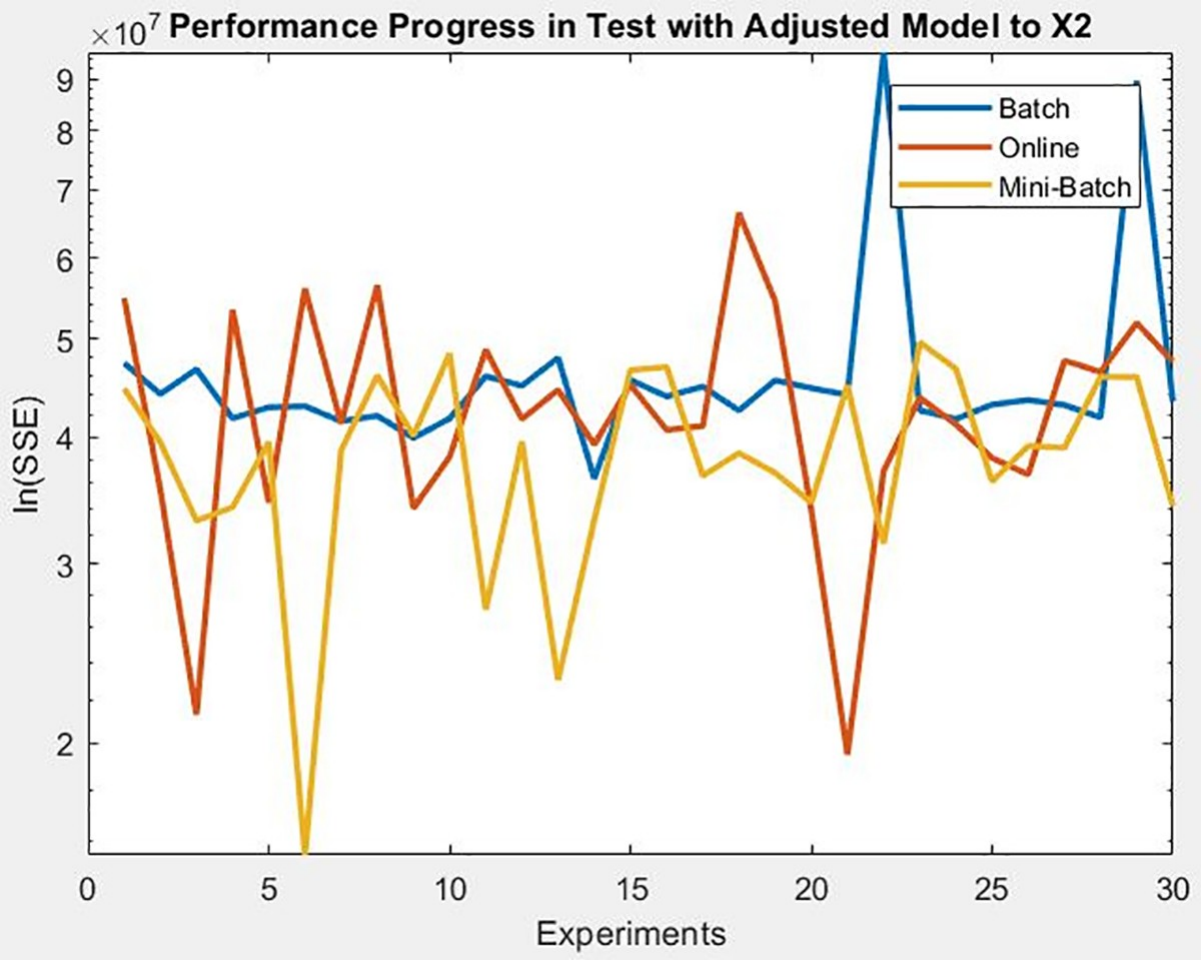
Performance progress of SEE in test stage with adjusted model by learning method to engine behavior dataset to output 2.

For the engine behavior dataset, the learning method that achieved a better minimization of the SSE during training and also during the test on the adjusted model was for mini-batch, as can be seen in Fig 25, Fig 26 and Fig 27 respectively, also supporting the conclusion of the mean and standard deviation results shown in Table 7.

**Fig 28:**
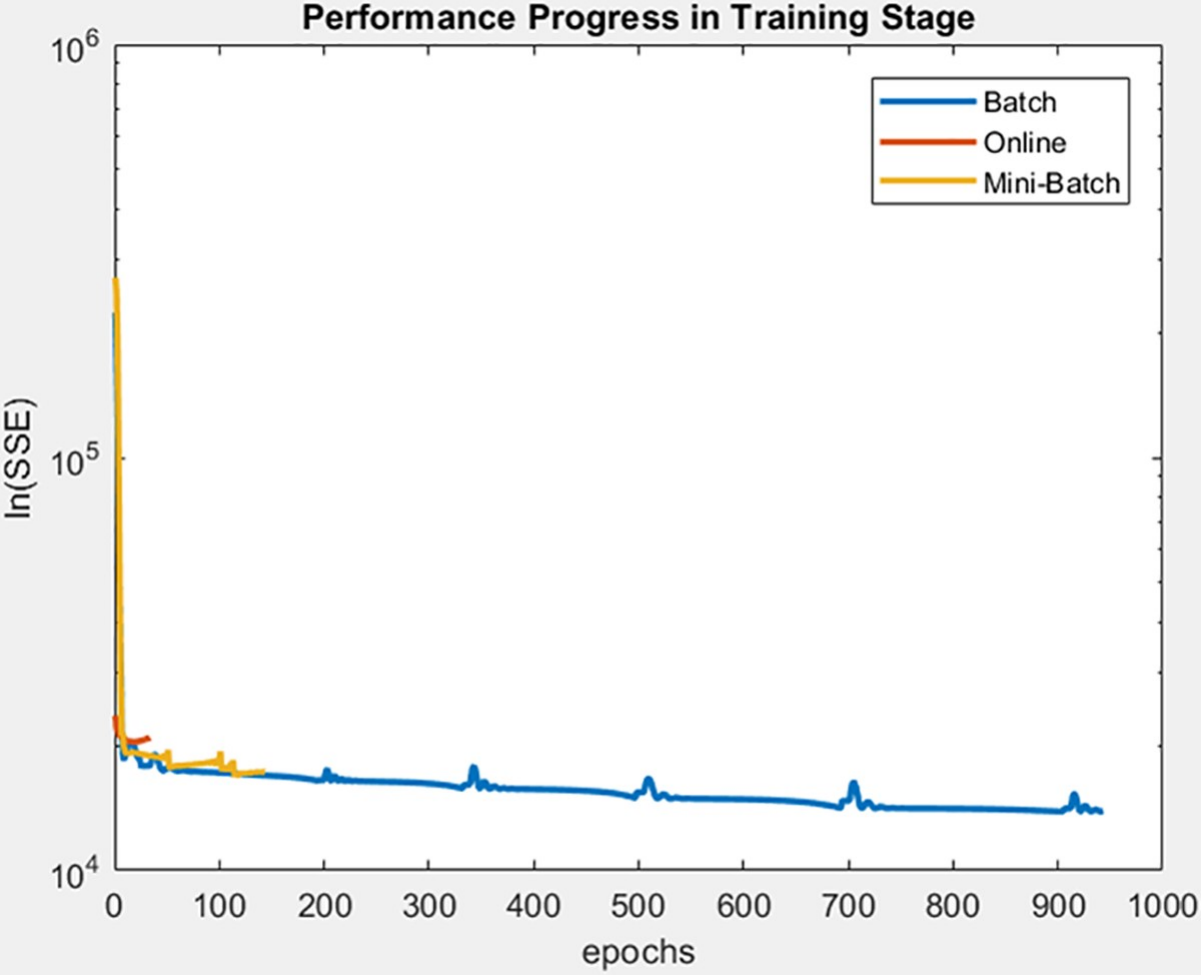
Performance progress of SEE in training stage by learning method to abalone shell rings dataset.

**Fig 29:**
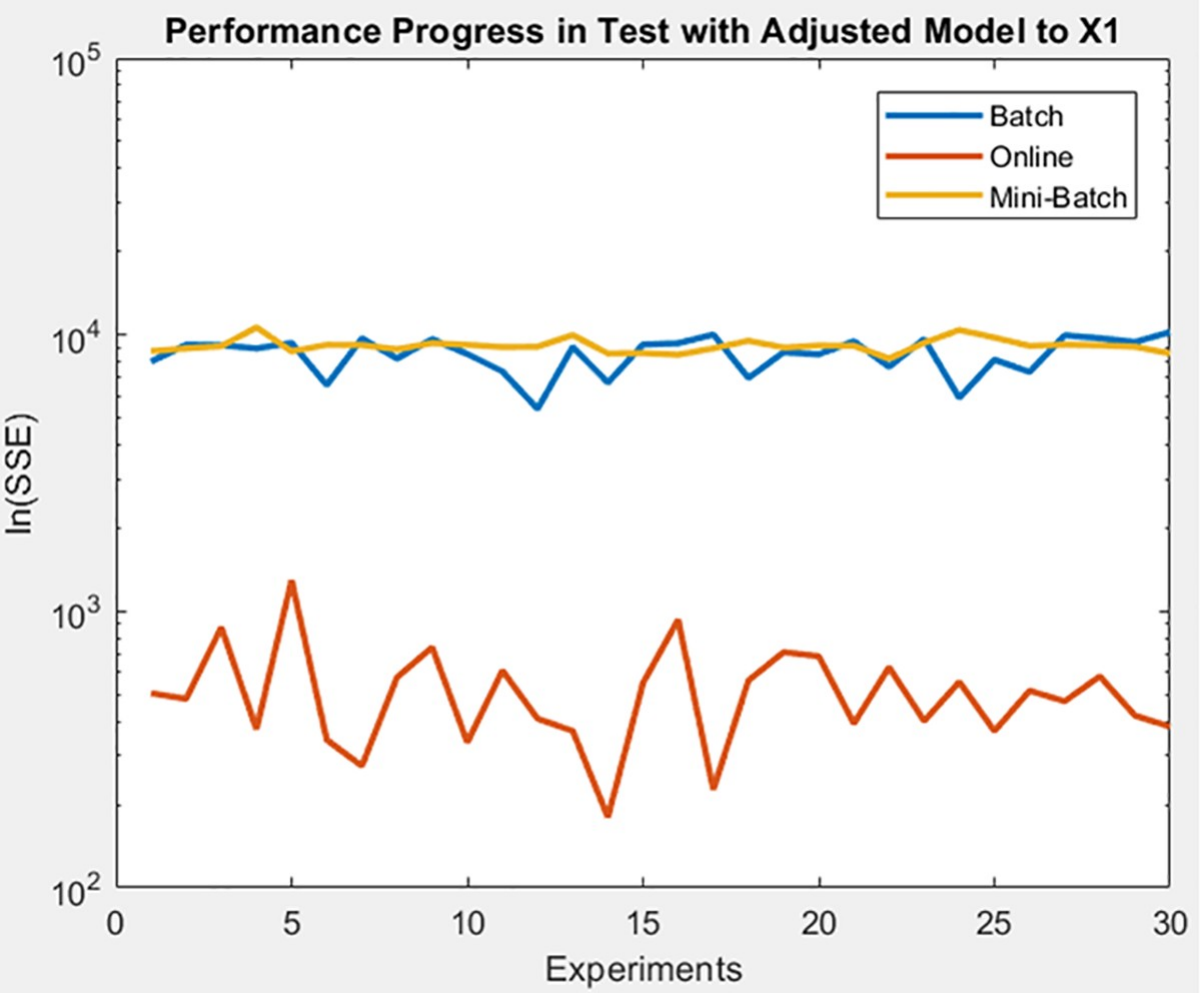
Performance progress of SEE in test stage with adjusted model by learning method to abalone shell rings dataset.

Finally, for the abalone shell rings dataset, the learning method that achieved a better minimization of the SSE during training was for batch and during the test on the adjusted model it was for online, as can be seen in Fig 28 and Fig 29 respectively, also supporting the conclusion of the mean and standard deviation results shown in Table 7.

## Conclusions

This paper establishes the basis for the implementation of Full Batch, Online and Mini-Batch learning methods both in a manner theoretical and practical, those methods are oriented to the processing of data according to the size or volume of the sample data and the form that the gradient vector is built and the parameters are adjusted or updated. Due to existing neuro-fuzzy systems which can only be trained under full batch learning method, it was necessary to implement a Mamdani based Neuro-Fuzzy with Center-of-Sets Defuzzification with the flexibility to work with any of the three learning methods during the training stage.

The main contribution of this paper as a first approach was to offer detailed theoretical and practical procedures about the three learning methods on a neuro-fuzzy system with the objective to better understand its functionality, performances, and behaviors under different contexts when a model is built. A variety of synthetic and real datasets with small and medium volume were used to carry out the experimentation.

The results obtained at a general level, that is, for each learning method evaluated, were based on the mean, obtained from all the experiments generated from different analyzed datasets through regression model built from neuro-fuzzy system proposed. It was observed that the Mini-Batch learning method with respect to the general trend of $\bar{R}$ was positive, It showed greater strength in its correlation and better control in its variability with mean results of 0.8268 y 14.12% respectively, followed by the Batch learning method with $\bar{R}$ = 0.7708 and finally the Online learning method $\bar{R}$ = 0.7520 but both with a very close coefficient of variation, with a difference of 0.58%, giving advantage to the Batch learning method. A similar behavior occurred with respect to the determination coefficient where the Mini-Batch learning method obtained a $\bar{R^{2}}=$ 0.7444 with a *C*.*V* = 6.50%, followed by the Batch learning method with $\bar{R^{2}}=$ 0.6906 and finally, the online learning method with $\bar{R^{2}}=$ 0.6456, with a *C*.*V* = 14.61% y 15.51% respectively. Finally, it is observed that the most stable learning methods in their experimental executions were the Batch and Mini-Batch learning method, being Batch learning method who had an advantage, but only by 1% more than Mini-Batch, it is also observed that it was these two learning methods that required the least number of rules, but it was the Mini-Batch and Online learning method that finished their training in fewer epochs.

We consider that the study is relevant due to the growing necessity of tools that have the possibility of being able to handle and analyze large volumes of data, and that allows for adequate management of their computational resources. The Mini-Batch learning method is highlighted as a very good alternative, since it can be performed in distributed environments because they are highly parallelizable and attenuate the difficulties that the Batch and Online learning methods present individually. As subsequent works, it is necessary to look for other methods of optimization that will lead us to better results with respect to the minimization of the error function and also to scale it to Deep Learning and Big Data environments, where treatment of high volumes of data are required for its processing.

## Supporting information

S1 Code (ZIP)
